# Non-canonical function of the splicing activator U2AF2 in promoting intron retention in the lncRNAs *PURPL* and *MALAT1*

**DOI:** 10.64898/2026.02.19.706780

**Published:** 2026-02-20

**Authors:** Ioannis Grammatikakis, Chosita Norkaew, You Jin Song, Amit K. Behera, Erica C. Pehrsson, Corrine Corrina R. Hartford, Shreya Kordale, Rishabh Prasanth, Yongmei Zhao, Biraj Shrethsa, Xiao Ling Li, Ravi Kumar, Ragini Singh, Tayvia Brownmiller, Xinyu Wen, Natasha J. Caplen, Pablo Perez-Pinera, Kannanganattu V. Prasanth, Thomas Gonatopoulos-Pournatzis, Ashish Lal

**Affiliations:** 1 Regulatory RNAs and Cancer Section, Genetics Branch, Center for Cancer Research (CCR), National Cancer Institute (NCI), National Institutes of Health (NIH), Bethesda, Maryland, USA; 2 Department of Cell and Developmental Biology, Cancer Center at Illinois, University of Illinois at Urbana-Champaign, Urbana, Illinois, USA; 3 Functional Transcriptomics Section, RNA Biology Laboratory, NCI, NIH, Frederick, Maryland, USA; 4 OMICS Technology Facility, Bioinformatics, Genetics Branch, CCR, NCI, NIH, Bethesda, Maryland, USA; 5 Advanced Biomedical Computational Science, Frederick National Laboratory for Cancer Research, Frederick, Maryland, USA; 6 University Laboratory High School, Urbana, Illinois, USA.; 7 Sequencing Facility Bioinformatics Group, Bioinformatics and Computational Science Directorate, Frederick National Laboratory for Cancer Research, NCI, NIH, Frederick, Maryland, USA; 8 Functional Genetics Section, Genetics Branch, CCR, NCI, NIH, Bethesda, Maryland, USA; 9 Department of Bioengineering, University of Illinois at Urbana-Champaign, Urbana, Illinois, USA

**Keywords:** U2AF2, lncRNA, *PURPL*, *MALAT1*, intron retention, splicing, CRISPR/Cas9, nuclear speckles, non-canonical

## Abstract

Intron retention (IR) is a form of alternative splicing in which an intron that is typically spliced out is retained in the mature RNA. Despite emerging evidence of widespread IR in protein-coding genes and lncRNAs, the underlying molecular mechanisms remain unclear. Here, we developed a genome-wide screen termed CRASP-seq, to investigate the mechanisms underlying IR in the lncRNA *PURPL.* Unexpectedly, the top hit was the essential splicing activator U2AF2 that promoted IR in *PURPL* by directly binding to a weak polypyrimidine tract. U2AF2 promoted IR in additional transcripts, including the nuclear speckle-localized *MALAT1* whose localization to nuclear speckles was disrupted upon U2AF2 depletion. Importantly, retention of a specific endogenous *MALAT1* intron was critical for its nuclear speckle localization and promoting cell migration. These findings uncover a non-canonical function of U2AF2 in promoting IR and reveal how IR contributes to the subcellular localization and functions of *PURPL* and *MALAT1*.

## Introduction

Splicing refers to the process of intron removal and exon “stitching” as part of RNA processing that occurs in the nucleus and is catalyzed by a large nucleoprotein complex called the spliceosome.^1-3^ Splicing is a highly regulated process where *cis*- and *trans*-acting factors coordinate proper and efficient spliceosomal recruitment.^4^ Several consensus sequences in the RNA are crucial for splicing: the 5′ and 3′ splice sites (ss), which demarcate the exon-intron boundaries of the intron to be removed, typically conform to the consensus sequence of GU-AG. The polypyrimidine (Py)-tract is a stretch of pyrimidines close to the 3′ ss and the branch point that lies upstream of the Py-tract.^1^ The splicing factor U2AF1 (U2-auxiliary factor 1) binds to the 3′ ss and U2AF2 binds to the Py-tract. The U2AF heterodimer, along with SF1 (Splicing factor 1), is recruited to the branch point to form the pre-spliceosomal E complex. This complex serves as a critical intermediate for the subsequent assembly of the spliceosome and splicing catalysis.^5-9^

The vast majority of human genes encode pre-mRNAs that contain introns that are spliced out and most nascent transcripts undergo alternative splicing, which leads to the combinatorial joining of exons. Alternative splicing is a major contributor to protein isoform diversity where multiple transcript variants can be produced from a single gene.^10-12^ There are several types of alternative splicing such as exon skipping, intron retention, mutually exclusive exons, and alternative 5′ and 3′ ss.^13^ One of the least studied forms of alternative splicing is intron retention (IR). Lately, it has drawn more attention since it has an important role in post-transcriptional gene expression regulation, and it is believed that more than half of human multi-exonic genes are affected by IR.^14^ IR frequently leads to nuclear detention of the transcripts and eventually degradation which may serve as a defense mechanism of the cell against deleterious products. The extra sequence added by the retained intron may change the open reading frame (ORF) of protein-coding genes and lead to nonsense-mediated decay (NMD)^15-17^ or initiate the use of an upstream ORF.^18,19^ In addition, they can contain binding sites for microRNAs (miRNAs) or RNA-binding proteins (RBPs), or they may form structural changes that impact the function of the transcript.^20^ Interestingly, IR has been associated with several diseases such as cancer where tumor cells show higher levels of IR compared to normal tissues.^21^ Studies on IR have shown that retained introns have been associated with weaker (non-consensus) splice sites, higher GC content, various lengths of introns, and higher intronic sequence conservation.^14,22^ However, the molecular mechanisms of IR regulation remain largely elusive.

Long non-coding RNAs (lncRNAs) are RNA molecules >200 nucleotides (nt) long that have no obvious coding potential. Similar to protein-coding genes, lncRNAs are transcribed by RNA Polymerase II, are 5′-capped, spliced and polyadenylated.^23^ However, several characteristics distinguish lncRNAs from protein-coding genes, including higher tissue-specific expression, lower abundance and stability, predominant nuclear localization, and having fewer but longer exons.^24-28^ One of the most distinguished features of lncRNAs is that they are less efficiently spliced compared to mRNAs.^26,29,30^ Interestingly, lncRNAs contain introns with much slower intron-excision kinetics compared to protein-coding genes.^31^ However, the regulatory mechanisms governing lncRNA splicing remain poorly understood.

lncRNAs have been shown to participate in splicing regulation, with *MALAT1* as the most prominent example. *MALAT1* is a highly abundant nuclear lncRNA which has oncogenic activity and is one of the first lncRNAs associated with cancer.^32-34^ It also has the ability to regulate alternative splicing because it resides in the nuclear speckles and can interact with several SR (Serine/Arginine rich) proteins to regulate their phosphorylation state and distribution between transcription sites and speckles.^33,35-37^ Nuclear speckles are sub-nuclear compartments that act as hubs in coordinating transcription, RNA processing and mRNA export.^38^ Several RBPs reside in nuclear speckles such as the protein SON which is a key component and marker of nuclear speckles.^39^ However, *MALAT1* itself is not essential for the structural integrity of nuclear speckles.^40,41^ Nevertheless, it remains unclear whether *MALAT1* undergoes alternative splicing and how this process impacts its localization to nuclear speckles.

Another lncRNA that we previously characterized and termed *PURPL* is transcriptionally induced by p53.^42^ *PURPL* plays a growth-promoting role in colorectal and liver cancer cells by distinct mechanisms including suppressing p53 protein levels. Interestingly, through transcriptomic analyses with both long-read and short-read sequencing, we identified multiple *PURPL* isoforms, several of which retain intron 2.^43^ However, the molecular mechanism governing *PURPL* intron 2 splicing has yet to be elucidated.

Here, we developed a novel CRISPR-based screening approach using a genome-wide guide RNA library and a minigene reporter containing the sequence of *PURPL* intron 2 and its flanking exons. This approach unexpectedly discovered a direct role of the splicing activator U2AF2 as a key factor promoting *PURPL* IR, contrary to its canonical function in facilitating intron removal. U2AF2 was similarly found to promote an intron retention program that includes two *MALAT1* introns. Interestingly, depletion of U2AF2 resulted in *MALAT1* exclusion from nuclear speckles. *MALAT1* knockout cells exogenously expressing *MALAT1* truncation mutants lacking specific introns identified one intron essential for the nuclear speckle association of the *MALAT1* transcript. Breast cancer cells where this intron is deleted showed reduced cell migration implying a functional role of the intronic sequences. These findings provide mechanistic insights into the splicing regulation of *PURPL* and *MALAT1*, uncovering a previously unrecognized non-canonical function of U2AF2 in promoting intron retention.

## Results

### A CRISPR-based screen identifies U2AF2 as an activator of *PURPL* intron 2 retention

**Figure 1.**
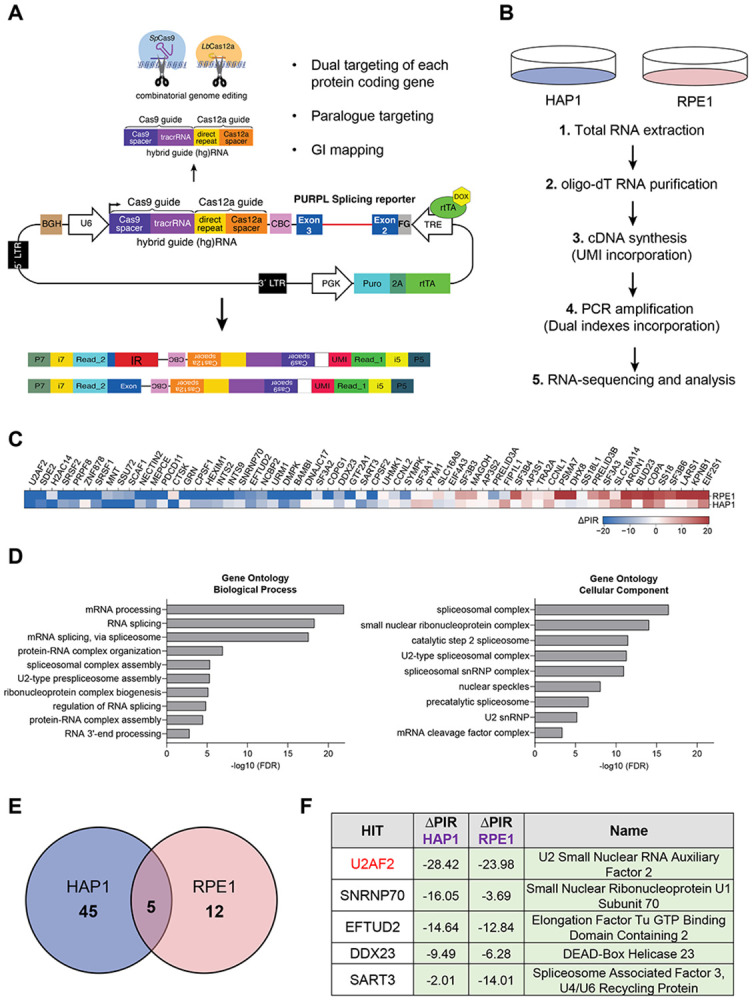
CRISPR-based screen identifies U2AF2 as a promoter of intron retention. **(A)** Schematic of the *PURPL* IR reporter used in the screen to identify IR regulators. The pLCHKO vector contains the puromycin resistance gene used for selection. The transgene also contains the genomic sequences of exon 2 (161 bp), intron 2 (1,943 bp), and exon 3 (163 bp) of the *PURPL* gene under a Doxycycline-inducible promoter. A hybrid guide (hg)RNA for guiding SpCas9 and LbCas12a is expressed under a U6 promoter. A construct with a hgRNA targeting an intergenic region was used as a negative control. Top arrow: The hgRNA is cleaved by Cas12a to produce 2 guide RNAs for dual targeting of each protein coding gene, paralog targeting, and genetic interaction mapping. Bottom arrow: Upon intron splicing or intron retention, two different reads are generated. **(B)** Two different cell lines, stably expressing Cas9 and Cas12a, are used: HAP1, and RPE1. The pipeline before RNA-seq is depicted. **(C)** Heatmap showing single genes only (after removing gene pairs), whether identified individually or as part of a gene pair. The hits are ordered by the average ΔPIR values across HAP1 and RPE1, from lowest to highest. **(D)** Left: Gene ontology enrichment analysis of biological processes regulated by the hits of the screen. The most significant pathways involve mRNA processing and RNA splicing. Right: Gene ontology enrichment analysis of cellular components regulated by the hits of the screen. These components include mainly Spliceosomal Complex, small nuclear ribonucleoprotein complex and the Catalytic step 2 Spliceosome. **(E)** Venn diagram showing that there are 5 common hits which cause a reduction in the percentage of intron retention (ΔPIR) of the splicing reporter in the two cell lines. **(F)** Table showing the top 5 hits of the screen with a ΔPIR for each hit in each cell line and their full names.

Previously, we identified *PURPL* as a p53-regulated lncRNA that promotes the growth of colorectal and liver cancer cells.^42,43^ In those studies, we reported that *PURPL* undergoes alternative splicing, and interestingly intron 2 of *PURPL* which is ~1.9 kb long, was either retained or spliced in the mature transcript.^43^ To identify proteins that regulate *PURPL* intron 2 retention, in the present study we used a CRISPR-based screening methodology that we recently developed, termed CRISPR-based identification of Regulators of Alternative Splicing with Phenotypic Sequencing (CRASP).^44^ This approach utilizes a genome-wide knockout lentiviral library targeting ~18,500 protein-coding genes using a dual hybrid guide (hg)RNA system. This system combines *Streptococcus pyogenes* (*Sp*)Cas9 and optimized *Acidaminococcus sp.* (op)Cas12a nucleases, where the hgRNA is cleaved by the intrinsic RNase activity of Cas12a to produce two guide RNAs for dual targeting in a given cell.^45-48^ The library also includes hgRNAs targeting paralogs (~2,400 pairs), and a curated set of genetic interaction pairs (~500), enabling combinatorial gene targeting from a single construct. Each gene and gene pair are targeted by four independent hgRNAs. Additionally, the library includes ~500 intergenic and non-targeting controls, totaling around 91,000 hgRNAs. Importantly, the lentiviral library incorporates a *PURPL* IR reporter expressed under a doxycycline-inducible Tet Response Element (TRE). The *PURPL* minigene reporter is comprised of the full native intron 2 sequences (~1.9 kb) flanked by the adjacent exons 2 (161 bp) and 3 (163 bp) (Figure 1A).

We performed the CRASP-screen in two cell lines: HAP1 and RPE1. Following puromycin selection for 48 h and doxycycline induction for an additional 24 h, RNA was extracted, and poly-A RNA was purified using oligo(dT) beads to exclude transcripts still undergoing processing. cDNA was synthesized using customized primers containing unique molecular identifiers (UMIs), facilitating the removal of PCR duplicates during downstream analysis. The synthesized cDNA was then utilized to construct Illumina sequencing libraries for paired-end sequencing. This sequencing approach enabled simultaneous quantification of transcripts that either retain or splice out the intron of interest from one read, while the corresponding hgRNA sequences were captured in the other read to pinpoint specific genetic perturbations. By directly linking *PURPL* splicing outcomes with genetic modifications introduced by each hgRNA in the library, this platform allowed the genome-wide exploration of *PURPL* splicing regulators (Figure 1B).

We employed an analytical pipeline that prioritized candidate genes based on multiple hgRNAs showing significant deviations from intergenic controls, with consistent identification across two technical replicates. This analysis identified a total of 62 genes that influence *PURPL* intron 2 splicing, the majority of which act as suppressors of intron 2 splicing (Figure 1C and Table S1). Gene ontology enrichment analysis of biological processes revealed a strong enrichment for terms related to mRNA processing and RNA splicing. Additionally, analysis of cellular components indicated that the identified hits are predominantly associated with Spliceosomal Complex, including the U2-type Spliceosomal Complex and the Catalytic step 2 Spliceosome (Figure 1D). These findings validate the effectiveness of our screening approach.

Consistent with the endogenous *PURPL* transcript isoforms, the basal levels of IR of the reporter were very high, with the majority of amplified transcripts corresponding to intron-containing isoforms, as expected based on the intensity of the Illumina libraries before sequencing (Figure S1A). Thus, the detection of genes that suppress IR might be below the detection levels of this screen. Therefore, we focused on the genes that promote intron retention. By calculating the change in Percent Intron Retention (ΔPIR = PIR_guide_ – PIR_intergenic_) across the two cell lines, we identified 5 unique genes as regulators of *PURPL* intron 2 retention, all of which act to suppress intron 2 splicing (Figure 1E and Table S1). The small number of shared hits between the two cell lines primarily reflects the stringent hit-calling criteria (odds ratio = 204.4, p=2.81xe^−10^) and cell-type-specific editing constraints.

Surprisingly, the gene with the strongest impact based on the average ΔPIR metric and identified as a hit in both cell lines, was U2AF2 (Figure 1F). This was followed by Small Nuclear Ribonucleoprotein U1 Subunit 70 (SNRNP70), a core component of the U1 snRNP, and Elongation Factor Tu GTP Binding Domain, which is associated with U5 snRNP (Figure 1F). Our finding that U2AF2 was promoting IR of *PURPL* intron 2 was unexpected because U2AF2 is a component of the spliceosome machinery, and its canonical function is to promote intron removal.^5,9^ RT-PCR assays confirmed our findings, showing that HAP1 and RPE1 cells transduced with *PURPL* minigene reporter constructs exhibit a striking increase in intron 2 splicing when U2AF2-targeting hgRNAs are expressed, relative to cells expressing hgRNAs targeting intergenic regions, which served as controls. When the control hgRNAs are used, *PURPL* intron 2 is mostly retained (Figure S1B). Our results suggest that depletion of U2AF2 results in decreased *PURPL* intron 2 retention, contradictory to its canonical function.

### U2AF2 binds to *PURPL* intron 2 transcripts and promotes intron retention

**Figure 2.**
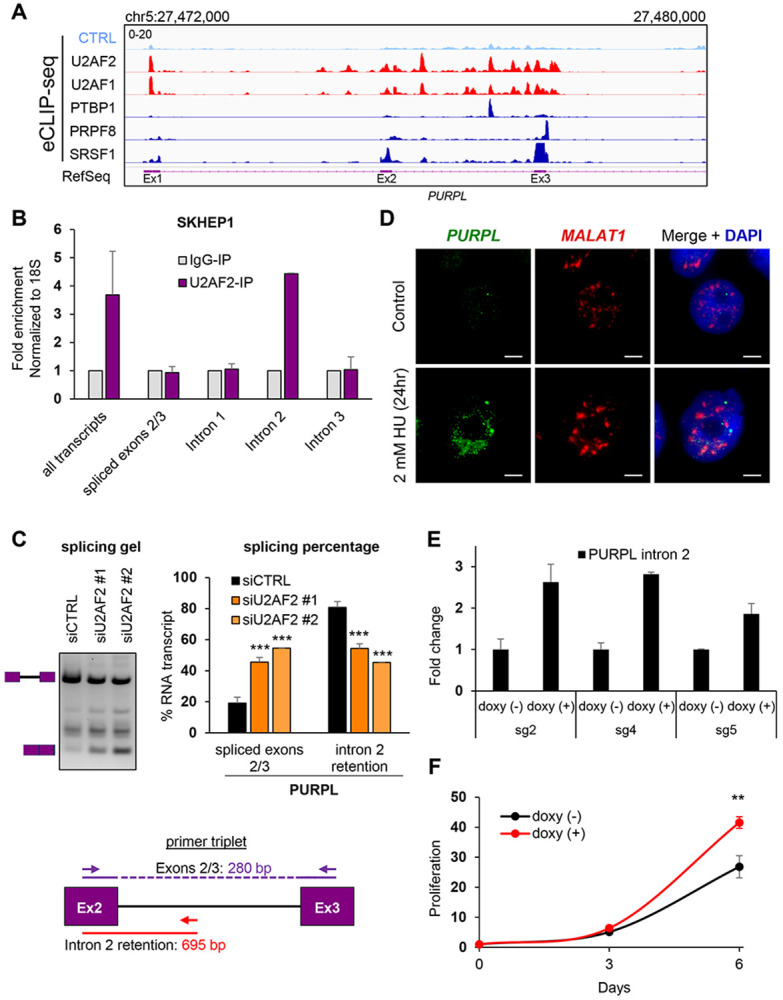
U2AF2 binds to intron 2-containing *PURPL* transcripts and promotes intron 2 retention. **(A)** IGV snapshot of eCLIP-seq data showing binding sites and enrichment of U2AF2 and U2AF1 on the *PURPL* transcripts around intron 2. eCLIP-seq data for PTBP1, PRPF8, and SRSF1 are indicated. The annotated locus by RefSeq is also indicated. eCLIP data-seq was downloaded from encodeproject.org. **(B)** RT-qPCR after RNA-IPs using a U2AF2 antibody with primer pairs specifically detecting *PURPL* transcripts as indicated in Figure S1C. U2AF2 binds to transcripts containing intron 2 but not the ones with intron 1, intron 3, or spliced exons 2 and 3. Samples were normalized to IgG-IP. 18S was used as a loading control. **(C)** Top panel: Gel with RT-PCR products for *PURPL* upon knockdown of U2AF2 with 2 different siRNAs in SKHEP1 cells. The schematics next to the gel indicate the expected products of the intron-retained and spliced isoforms. Between the two expected PCR products, we observed an extra band corresponding to the inclusion of an alternative exon inside *PURPL* intron 2 as observed in RefSeq, the inclusion of which is not affected by U2AF2. Quantitation of the gel bands is shown in the graphs on the right. Bottom panel: Schematic of the PCR primer triplet used to detect intron 2 retention (red) or splicing (purple). The length for each PCR product is indicated. **(D)** RNA-FISH images for *PURPL* with intron 2 retention and *MALAT1* in HCT116 cells without treatment or after 24 hr of 2 mM of Hydorxyurea (HU) to induce *PURPL* expression. Scale bar is 10μm. **(E)** RT-qPCR for intron 2-containing *PURPL* transcript after 48 hr of 1μg/ml doxycycline treatment in comparison to no treatment in SKHEP1 *PURPL*-CRISPRi populations using 3 different gRNAs. **(F)** Proliferation assay showing the effect of overexpression of intron 2-containing *PURPL* transcript in the proliferation of SKHEP1 cells where the endogenous *PURPL* is knocked down with CRISPRi. The graph depicts the average of 3 populations with different gRNAs. The cells were treated with 1ug/ml doxycycline to induce intron 2-retained *PURPL* expression and cell proliferation was monitored at 3 and 6 days. Error bars represent standard deviations from 2 **(E,)** and 3 **(C)** experiments. **p<0.01, ***p<0.001.

U2AF2, as a heterodimer with U2AF1, binds to the Py-tract and the 3′ ss, respectively, to promote splicing.^5,8^ To elucidate its role in *PURPL* splicing, we investigated whether U2AF2 binds to intron 2. Enhanced Crosslinking and Immunoprecipitation sequencing (eCLIP-seq),^49^ from the ENCODE project^50,51^ from HepG2 cells revealed multiple U2AF2 and U2AF1 binding sites not only proximal to the 3′ ss of *PURPL* intron 2 but also broadly distributed across intron 2 (Figure 2A). Compared to eCLIP data for other RBPs, U2AF1 and U2AF2 showed the highest density of binding sites and the most prominent peaks. Specific binding of U2AF2 to intron 2 was confirmed by performing RNA immunoprecipitation (RNA-IP) using a U2AF2-specific antibody and primer pairs designed to detect *PURPL* transcripts (Figure S1C). U2AF2 was found to interact with transcripts containing *PURPL* intron 2, but not with the spliced *PURPL* transcripts (Figure 2B).

To functionally validate the role of U2AF2 in *PURPL* intron 2 retention, we performed RT-qPCR to quantify endogenous *PURPL* transcript isoforms upon U2AF2 knockdown using two independent siRNAs (Figure S1D). This analysis revealed an increase in the spliced isoform and decrease in *PURPL* intron 2 retention in SKHEP1 cells (Figure S1E), as well as other cell lines such as HepG2 and U2OS cells (Figures S2A and S2C). To further validate the role of U2AF2 on *PURPL* intron 2 splicing, we also conducted semi-quantitative RT-PCR assays from SKHEP1, HepG2, and W138 cells using a primer triplet (Figure 2C, S2B and S2D). In all cell lines tested, U2AF2 depletion led to an increase in *PURPL* intron 2 splicing, consistent with findings from the minigene reporter assay and orthogonal CRISPR-mediated U2AF2 knockdown experiments.

It should be noted that although U2AF2 knockdown did not completely abolish intron retention, the effect of U2AF2 knockdown was reproducible and increased splicing of *PURPL* intron 2 from ~20% to ~40% across multiple cell lines, representing a highly significant change that effectively doubled the abundance of the spliced *PURPL* isoform. There are several plausible explanations for the partial nature of this effect. First, because U2AF2 is an essential gene, we were unable to perform multiple rounds of siRNA transfection, which likely limited the extent of downstream effects upon U2AF2 knockdown that could be achieved without compromising cell viability. Consistent with this, as shown in Figure S1B, when a sgRNA was used to target U2AF2 we observed a strong reduction in *PURPL* intron 2 retention and increased splicing in both HAP1 and RPE1 cells. Second, *PURPL* intron retention may be regulated by additional RNA-binding proteins in addition to U2AF2 as revealed by our CRISPR-screen (Figure 1F). In such a combinatorial regulatory context, depletion of U2AF2 alone would be expected to produce only a partial reduction in intron retention. Together, these observations support a significant and biologically meaningful role for U2AF2 in promoting *PURPL* intron 2 retention despite the incomplete penetrance of the knockdown.

Given that U2AF2 functions as a heterodimer with U2AF1, we also knocked down U2AF1 in SKHEP1 cells and observed a similar effect of U2AF2 knockdown on *PURPL* intron 2 retention regulation (Figures S2E and S2F). We would expect that U2AF1 would also come as one of the top hits in the screen but U2AF1 gRNAs were not included in the library due to high off-target scores of the guides. These results demonstrate that the U2AF complex promotes *PURPL* intron 2 retention and that both U2AF1 and U2AF2 can promote intron retention.

### The intron-retained *PURPL* transcript is unstable and localizes to the nucleus

We previously observed an enrichment of the intron 2-retained *PURPL* transcript in the nucleus.^43^ To confirm this result, we conducted single molecule RNA-FISH in HCT116 cells using probes hybridizing to *PURPL* intron 2 (Figure 2D). Because *PURPL* is a known target of p53 and is upregulated upon DNA damage,^42^ we treated the cells with the DNA damaging agent hydroxyurea for 24 hr to induce *PURPL* expression. RNA-FISH confirmed the exclusive nuclear localization of the *PURPL* intron 2 containing transcript. To assay for subnuclear compartmentalization, we also performed RNA-FISH to detect *MALAT1*, which resides in nuclear speckles.^36^ Intron 2-retained *PURPL* transcripts did not colocalize with *MALAT1* indicating that it does not localize to nuclear speckles (Figure 2D). We further validated this result by conducting nuclear/cytoplasmic fractionation assays followed by RT-qPCR showing that intron 2-containing transcripts are almost exclusively nuclear (Figure S3A).

Intron retention can affect transcript stability, with longer RNA species often being more vulnerable to degradation. To test if *PURPL* intron 2 effects transcript turnover, we conducted RNA stability assays using the transcription inhibitor Actinomycin D (ActD) and observed that the intron 2-retained *PURPL* transcript is less stable and has a significantly lower half-life than the *PURPL* transcript in which intron 2 is spliced out (Figure S3B). To identify the functional impact of *PURPL* intron 2, we first used CRISPRi to generate *PURPL*-depleted SKHEP1 cells by targeting 3 independent sgRNAs to the p53-response element of the *PURPL* promoter.^43^ We observed >80% reduction in the levels of endogenous *PURPL* in these experiments (Figure S3C). We next stably transduced these *PURPL*-depleted cells with a lentivirus to express exogenous *PURPL* transcript specifically containing intron 2 under the control of a doxycycline (doxy)-inducible promoter and observed ~2-3-fold induction of the exogenous *PURPL* intron 2 containing transcript (Figure 2E). Because we and others have previously shown that *PURPL* is overexpressed in some cancers and *PURPL* is a pro-proliferative lncRNA,^42,43,52,53^ we next performed cell proliferation assays from the doxy-inducible cells. Our results show that the cells expressing the intron 2-contaning *PURPL* proliferate faster (~2-fold) as compared to the no-doxy cells, after 6 days of doxy treatment (Figure 2F). To make sure that the observed increase in proliferation upon expression of exogenous *PURPL* transcript containing intron 2 was not due to the spliced *PURPL* isoform, we performed semi-quantitative RT-PCR, and as shown in Figure S3D, upon doxy treatment, only the intron retained *PURPL* transcript was detected. Importantly, as a negative control, there was no effect of doxy treatment on the proliferation of parental SKHEP1 cells (Figure S3E). Induction of intron 2-contaning *PURPL* transcripts did not have any effect in live cell percentage which remained at ~100% (Figure S3F). Taken together, these findings suggest that intron 2 retention affects *PURPL* by sequestering the transcript in the nucleus, reducing its stability and this transcript promotes cell proliferation.

### The weak polypyrimidine-tract in *PURPL* intron 2 is important for IR regulation by U2AF2

U2AF2 binds to the Py-tract to initiate spliceosomal assembly leading to splicing and intron removal.^5^ Suboptimal splicing efficiency may be the result of multiple factors, including splice site strength and the Py-tract sequence composition. The Py-tract is a stretch of 15-20 pyrimidines (mostly Us) immediately upstream of the 3′ ss. We analyzed the Py-tract of *PURPL* intron 2 and noticed that it deviates from the consensus, containing numerous purines (Figure S4A). To investigate whether U2AF2 regulation on intron 2 retention relies on the Py-tract sequence, we used the *PURPL* intron 2 minigene reporter system (Figure 1A). We mutated the Py-tract, replacing it either with a strong Py-tract from *PURPL* intron 1 or with a stretch of Us, simulating a strong Py-tract. U2AF2 was knocked down in SKHEP1 cells stably expressing the mutated Py-tract constructs and RT-PCR was conducted to monitor intron 2 splicing. Interestingly, the basal levels of intron retention were very different with different mutants; ~90% in intron 2 WT, ~30% in *PURPL* intron 1 and ~20% in the All-U construct (Figure S4B and S4C). This shows that the Py-tract is indeed, at least partially, responsible for the inefficient intron 2 removal in *PURPL* (Figure S4C). Surprisingly, when the WT sequence was replaced with the strong Py-tract from intron 1 or with a stretch of Us, U2AF2 knockdown continued to promote splicing. Because the minigene reporter contained the mutant Py-tract along with the remaining endogenous sequences from *PURPL* intron 2, the modest increase in splicing observed for the mutants after *U2AF2* knockdown may reflect U2AF2 inhibitory binding at additional sites within *PURPL* intron 2 beyond the Py-tract (Figure 2A). It may be that these regions function as a weak Py-tracts, which are sufficient for U2AF2 binding but not strong enough to efficiently recruit the spliceosome or promote intron removal. These results indicate that the endogenous intron 2 element in *PURPL* is a non-canonical, low-affinity Py-tract, and that its weakness may contribute to the high intron retention observed in *PURPL*.

### U2AF2 directly regulates multiple IR events including *MALAT1*

To determine if U2AF2 promotes IR in additional genes, we next performed total RNA-seq and PacBio Iso-seq from SKHEP1 cells transfected with siCTRL or siU2AF2. Consistent with our data, U2AF2 knockdown resulted in a marked increase in the splicing efficiency of *PURPL* intron 2 (Figures S5A and S5B). Additionally, we observed that U2AF2 depletion increased overall *PURPL* expression, with more reads mapping to all exons (Figures S5A and S5B and Tables S3 and S4).

**Figure 3.**
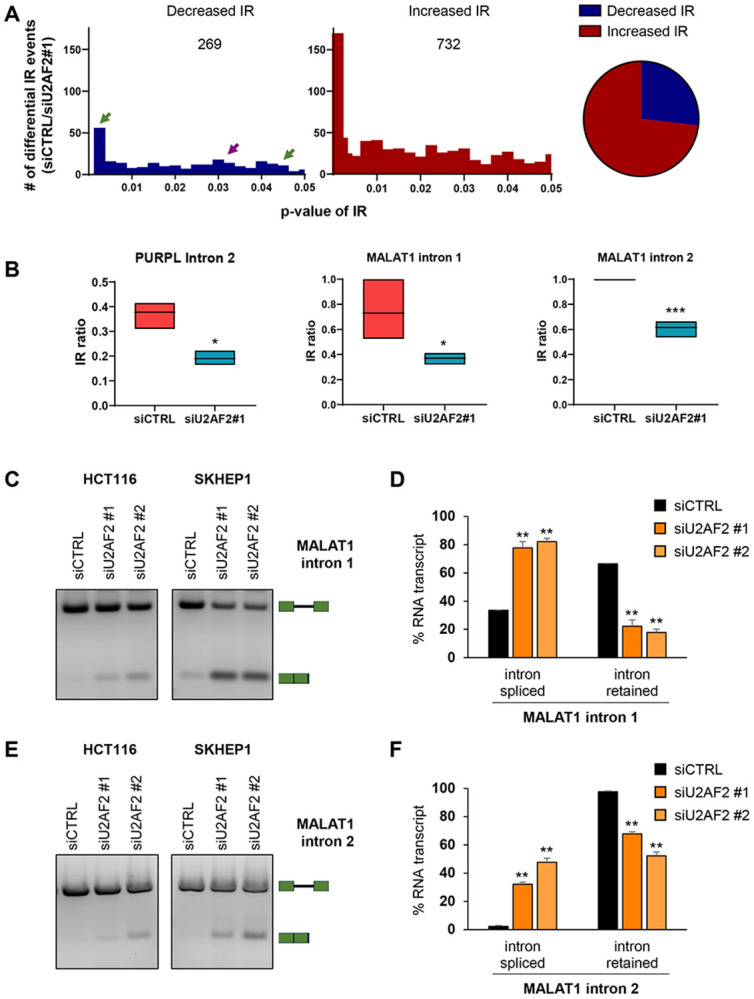
U2AF2 directly promotes Intron Retention of *MALAT1*. **(A)** U2AF2 was knocked down in SKHEP1 cells and 72 hr later, RNA was extracted and RNA-seq was performed. *Left*: Number of decreased (blue) and increased (red) IR events at various p-values after U2AF2 knockdown as analyzed with the IR Finder algorithm. The purple arrow indicates the *PURPL* IR event and green arrows indicate MALAT IR events *Right*: Pie chart of the numbers of increased and decreased IR events upon U2AF2 knockdown. **(B)** Floating bar plot showing the IR ratio of *PURPL* intron 2 and the IR ratio of intron 1 (middle) and intron 2 (right) of *MALAT1*. siCTRL and siU2AF2#1 samples as analyzed with the IRFinder algorithm. **(C)** and **(E)** RT-PCR for *MALAT1* using a primer pair flanking the regulated intron 1 **(C)** or intron 2 **(E)** upon knockdown of U2AF2 with 2 different siRNAs in HCT116 and SKHEP1 cells. The schematics next to the gel indicate the expected products of the intron-retained and spliced isoforms. **(D)** and **(F)** Bar graph with quantitation of the gel bands from **(C)** and **(E)** in SKHEP1 cells. Error bars represent standard deviations from 2 independent experiments. *p<0.05, **p<0.01, ***p<0.001.

To identify IR events regulated by U2AF2 at a transcriptome-wide level, we utilized IRFinder, an algorithm specifically designed to calculate changes in IR.^54^ This analysis revealed ~1000 statistically significant IR events regulated by U2AF2. For the majority of these events (732), there was an increase in the IR ratio (Intronic abundance/(Intronic abundance + exonic abundance)) after U2AF2 depletion showing that U2AF2 predominantly promotes splicing, as expected. However, in 269 events, U2AF2 knockdown resulted in decreased IR, indicating that U2AF2 promotes IR in a subset of genes (Figure 3A and Table S2). We next analyzed ENCODE U2AF2 eCLIP-seq data from HepG2 cells to determine if U2AF2 directly binds to the regulated transcripts. We found that in 96 out of the 269 events, U2AF2 was directly bound to the RNA, implying a direct intron retention regulation by U2AF2 (Figure S5C and Table S2).

To validate these findings, we selected three of the identified introns from *MDM1*, *RETREG2*, and *TMEM41B* (Figure S6A). We observed multiple U2AF2 binding peaks within the regulated introns from *MDM1* (Figure S6B). We next assessed their splicing in SKHEP1 cells following U2AF2 knockdown using two independent siRNAs. In all three cases, U2AF2 depletion significantly enhanced intron removal (Figures S6C, S6D, and S6E). IRFinder validated that *PURPL* intron 2 is one of the events showing a significant decrease in IR ratio upon U2AF2 depletion (Figure 3B).

Among the genes in which IR is promoted by U2AF2, there were only a few lncRNAs other than *PURPL*. One of the lncRNAs was *MALAT1*. IRFinder identified two introns in *MALAT1* that have weak Py-tract sequences similar to *PURPL* intron 2 (Figure S7). For both these introns in *MALAT1*, U2AF2 knockdown resulted in a significant decrease in the IR ratio compared to siCTRL (Figures 3A and 3B). This result was confirmed by RT-PCR from HCT116 and SKHEP1 cells upon U2AF2 knockdown (Figures 3C-F). These findings suggest that U2AF2 directly promotes IR in multiple transcripts through binding to the regulated introns. Similar to *PURPL*, depletion of U2AF2 does not result in complete loss of IR likely reflecting the fact that *U2AF2* is an essential gene which limited our ability to perform extended knockdown experiments without compromising cell viability. Additionally, depletion of U2AF2 alone may produce only partial effects when other factors contribute to the regulation of these introns.

### SON depletion rescues the effect of U2AF2 knockdown in *PURPL* intron 2 retention

**Figure 4.**
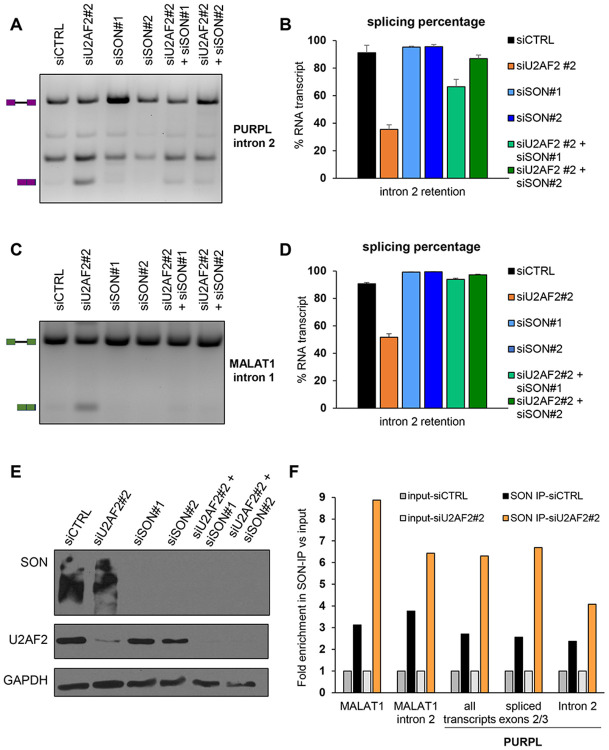
SON depletion rescues the effect of U2AF2 knockdown in *PURPL* and *MALAT1* intron 2 retention. **(A)** and **(C)** RT-PCR for *PURPL* **(A)** and *MALAT1* **(C)** transcripts upon knockdown of U2AF2, knockdown of SON or simultaneous knockdown indicating a rescue effect. The schematics next to the gel indicate the expected products of the intron-retained and spliced isoforms. **(B)** and **(D)** Quantitation of the gel bands from **(A)** and **(C)**. Error bars represent the SD of 2 experiments. **(E)** Western blot for SON (upper panel) and U2AF2 (middle panel) showing successful knockdown of SON and U2AF2. GAPDH was used as a loading control (lower panel). **(F)** RT-qPCR after RNA-IP using a SON antibody with primer pairs specifically detecting *MALAT1* or *PURPL* transcripts upon U2AF2 knockdown as indicated in Figure S1C. Transcript -specific primers for *MALAT1* are shown in Table S5. SON binds to spliced transcripts more strongly upon U2AF2 depletion. Samples were normalized to input. GAPDH was used as a loading control. The graph shows a representative of 2 replicates.

To investigate the mechanism by which U2AF2 inhibits the removal of introns in *PURPL* and *MALAT1*, we considered the possibility of U2AF2 preventing the recruitment of other splicing factors which function towards removal of introns when weak splice sites are present. SON is a large RS-like protein mainly located in nuclear speckles where splicing factors reside, and it has been shown to have the ability of removing constitutive introns with weak splice sites.^55^ Based on this, we knocked down SON with 2 different siRNAs in SKHEP1 cells either alone or with simultaneous U2AF2 knockdown (Figures 4A-D). When SON alone was depleted, the effect on intron retention was minimal. However, SON depletion rescued the effect of U2AF2 depletion (Figures 4A-E). This indicates that U2AF2 and SON compete for *PURPL* intron 2 and *MALAT1* intron removal. We next asked the question whether U2AF2 presence affects the binding of SON in *PURPL* and *MALAT1* precursor RNAs. To test this, we conducted RNA-IP using a SON antibody and followed by RT-qPCR using primers specifically detecting *PURPL* and *MALAT1* transcripts (Figure 4F). SON showed higher binding to *MALAT1* and *PURPL* transcripts after U2AF2 depletion compared to negative control. These data suggest that U2AF2 inhibits *PURPL* intron 2 and *MALAT1* splicing partly due to the inhibition of recruitment of SON.

### Intron 2 of *MALAT1* drives its localization to nuclear speckles

**Figure 5.**
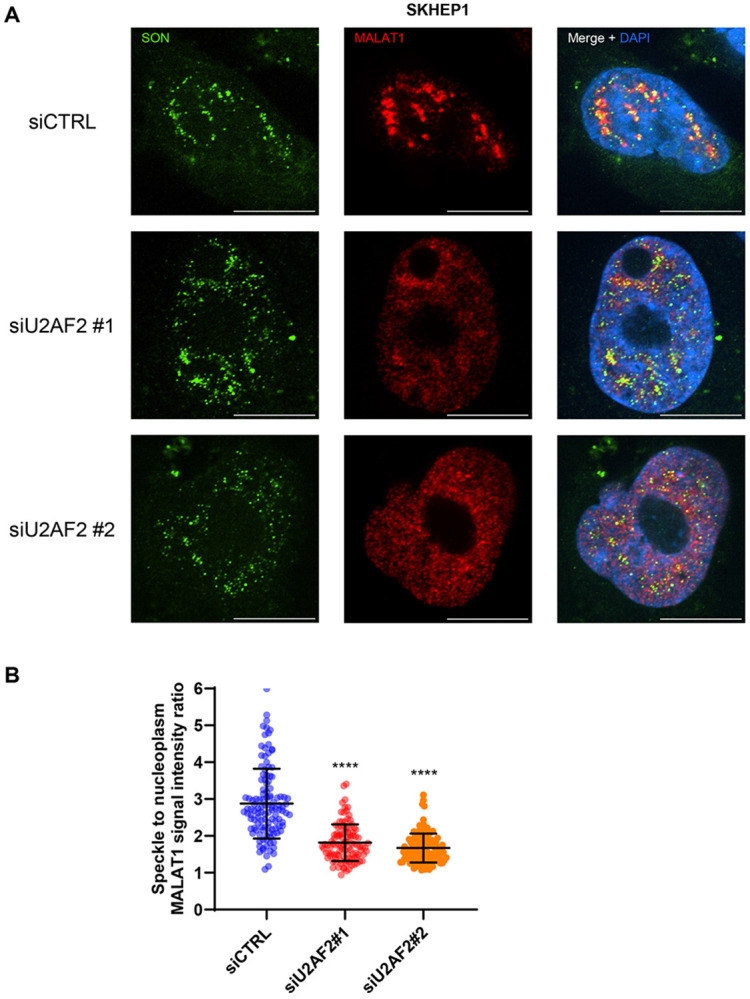
U2AF2 promotes localization of *MALAT1* to nuclear speckles. **(A)** RNA-FISH images for *MALAT1* and Immunofluorescence images for SON is shown upon transfection of SKHEP1 cells with siCTRL or siU2AF2. *MALAT1* is enriched in nuclear speckles in the siCTRL but not upon U2AF2 knockdown. **(B)** Quantitation of the speckle to nuclear plasma *MALAT1* signal ratio in the three replicates in panel **(A)**. ****p<0.0001.

*MALAT1* plays a role in regulating splicing by interacting with splicing factors in the nuclear speckles.^36^ To determine the impact of IR on *MALAT1* localization to nuclear speckles we performed RNA-FISH for *MALAT1* upon U2AF2 depletion with two different siRNAs in SKHEP1 and HCT116 cells (Figures 5A and S8A). Remarkably, U2AF2 knockdown resulted in a significant decrease in the ratio of *MALAT1* signal intensity in speckles relative to nucleoplasm (Figures 5A-B and S8A-B). U2AF2 depletion did not affect total *MALAT1* levels as observed in the RNA-seq results (Table S3). This reduction does not compromise the structural integrity of nuclear speckles as indicated by immunostaining for the RNA-binding protein SON, a well-established nuclear speckle marker^39^ (Figures 5A and S8A) and is consistent with previous reports showing that *MALAT1* is not essential for the integrity of nuclear speckles.^40,41^ Together, these results suggest that U2AF2 plays a role in promoting *MALAT1* localization to nuclear speckles.

To determine whether it is the intron retention in the transcript *per se* which directs *MALAT1* localization or if U2AF2 indirectly regulates its localization, we assessed the nuclear speckle localization of wild-type (WT) and intron-deleted *MALAT1* transcripts. To do this, we used CRISPR/Cas9 to generate two *MALAT1* knockout (KO) clones in HCT116 by inserting ~100 bp downstream of the transcription start site, a puromycin resistance gene in one allele and a hygromycin resistance gene in the other allele (Figure S9A). In both KO clones, there was >95% reduction in *MALAT1* RNA levels as compared to a wild-type clone (Figure S9A).

**Figure 6.**
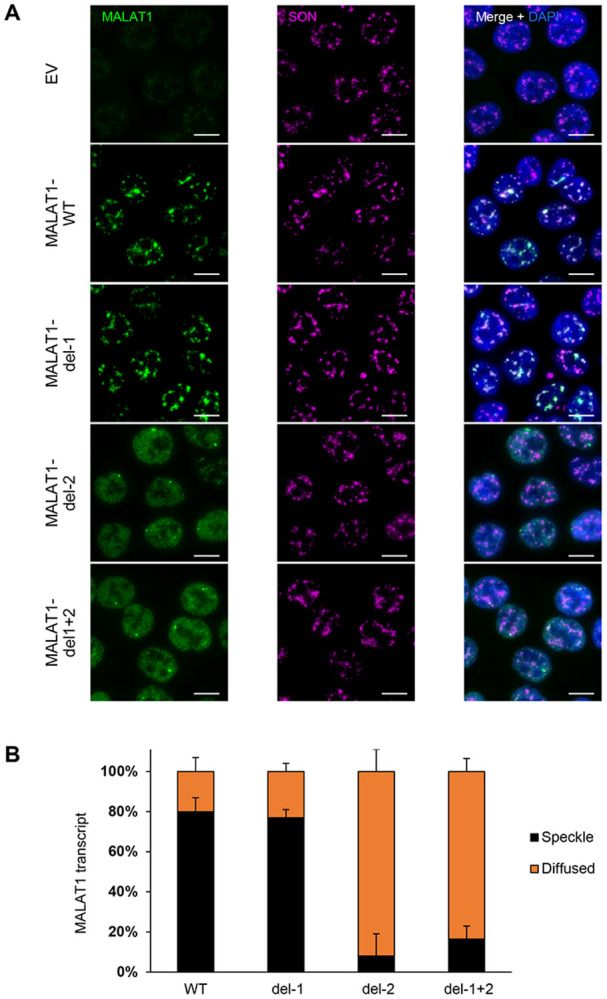
Intron 2 of *MALAT1* dictates its localization to nuclear speckles. **(A)** RNA-FISH images for *MALAT1* and Immunofluorescence images for SON in a HCT116 *MALAT1*-KO clone (clone 1) where Empty Vector (EV) or constructs that exogenously express *MALAT1* transcripts were re-introduced using a doxy-inducible lentivirus. Expressed *MALAT1* transcripts were full-length (WT) or intron 1 deleted (del-1) or intron 2 deleted (del-2) or both introns deleted simultaneously (del-1+2). The cells were first treated with 1ug/ml doxycycline for 48 hr to induce *MALAT1* expression. *MALAT1* without intron 2 does not enrich in speckles as it is shown with SON protein. Scale bar is 10μm. **(B)** Graph showing quantification of the percentage of cells where *MALAT1* transcripts are enriched in speckles or being diffused in HCT116 cells clone 1. Error bars represent standard deviations from 2 independent experiments.

Next, we transduced the *MALAT1* KO clones with an empty vector or doxycycline-inducible lentiviral constructs that exogenously express the full-length *MALAT1*-WT in which both introns are intact, intron 1 deleted *MALAT1* (del-1), intron 2 deleted *MALAT1* (del-2) or both introns were deleted (del-1+2). After 48 hours of doxycycline treatment to induce *MALAT1* expression, which was confirmed with RT-qPCR (Figure S9B), RNA-FISH combined with immunofluorescence for the nuclear speckle marker SON, revealed distinct localization patterns. As expected, exogenously expressed *MALAT1*-WT was predominantly (~80%) localized to nuclear speckles (Figures 6A and S10A). Similarly, *MALAT1*-del-1 was also predominantly (~80%) localized to nuclear speckles. Remarkably, *MALAT1* lacking intron 2 (del-2) or both introns (del-1+2) no longer localized (<15%) to nuclear speckles and showed diffused localization in the nucleus (Figures 6A and S10A). Importantly, this diffused localization upon intron 2 deletion was observed in both *MALAT1* knockout clones and was further confirmed through quantitative analyses (Figures 6B and S10B).

### Intron 2 of *MALAT1* promotes cell migration

**Figure 7.**
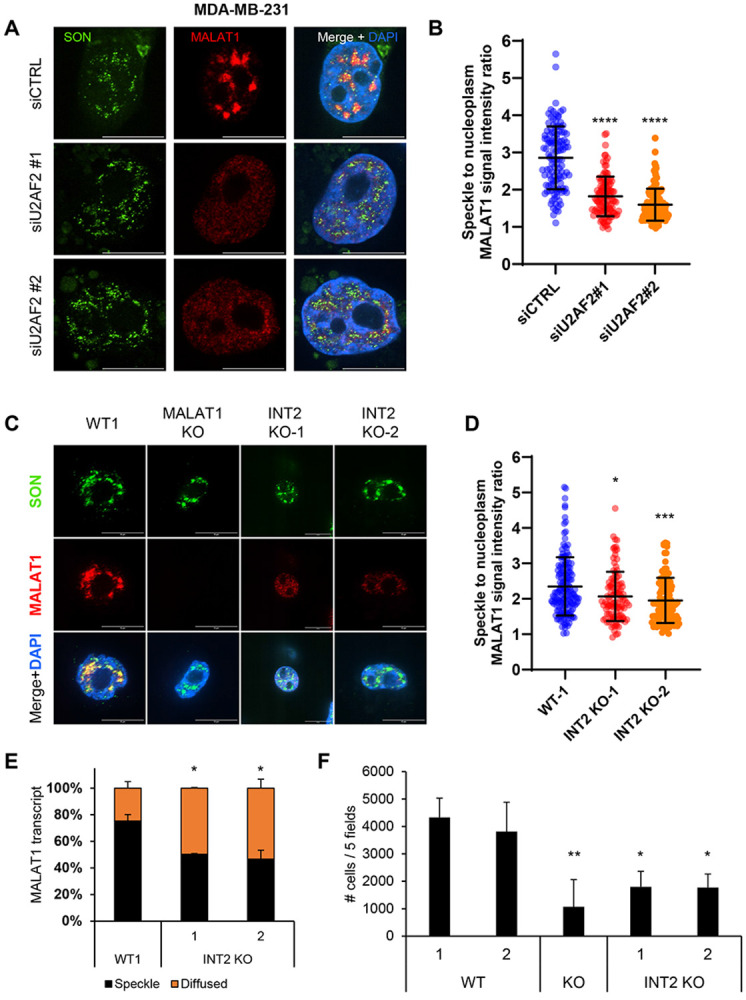
Intron 2 regulates the migration capacity of *MALAT1* in breast cancer cells. **(A)** RNA-FISH images for *MALAT1* and Immunofluorescence images for SON are shown upon transfection of MDA-MB-231 cells with siCTRL or siU2AF2. *MALAT1* is enriched in nuclear speckles in the siCTRL but not upon U2AF2 knockdown. **(B)** Quantitation of the speckle to nuclear plasma *MALAT1* signal ratio in the three replicates in panel **(A)**. **(C)** RNA-FISH images for *MALAT1* and Immunofluorescence images for SON in MDA-MB-231 clones either WT, or whole locus *MALAT1* deletion, or intron 2 deletion. When intron 2 is deleted, *MALAT1* does not enrich in speckles as it is shown with SON protein. Scale bar is 10μm. **(D)** Quantitation of the speckle to nuclear plasma *MALAT1* signal ratio in the three replicates in panel **(C)**. **(E)** Graph showing quantification of the percentage of *MALAT1* transcripts enriched in speckles or being diffused in MDA-MB-231 cells in panel **(C)**, N=2. **(F)** Bar graph showing the number of cells that have migrated in transwell migration assays conducted in MDA-MB-231 WT, *MALAT1* KO and *MALAT1* – intron 2 deletion clones. Intron 2 deletion leads to decreased migration potential. The number of cells are the sum of cells from 5 different fields, N=4. *p<0.05, **p<0.01, ***p<0.001, ****p<0.0001.

*MALAT1* has been shown to promote cell migration and metastasis^32,56^ and its expression has been correlated with high tumor progression and metastasis in various tumors.^57^ We therefore tested whether the intronic sequences that regulate *MALAT1* nuclear speckle localization are related to the ability of *MALAT1* to promote cell migration. To investigate this, we used the metastatic breast cancer cell line MDA-MB-231 and first performed RNA-FISH for *MALAT1* after knocking down U2AF2 with 2 different siRNAs. Similar to SKHEP1 and HCT116 cells, U2AF2 knockdown resulted in decreased localization of *MALAT1* to speckles in MDA-MB-231 cells (Figures 7A and B). We next utilized CRISPR/Cas9 to generate KO clones in MDA-MB-231 cells where either the whole *MALAT1* locus or intron 2 of *MALAT1* were deleted. *MALAT1* depletion in the *MALAT1* KO clone was confirmed with RT-qPCR (Figure S11A). RNA-FISH in 2 separate clones showed that intron 2 deletion reduced *MALAT1* localization to nuclear speckles compared to the WT clone (Figures 7C-E). In the intron-2 deletion clones, the transcripts containing intron 2 were, as expected, undetected with primers specifically amplifying intron 2. Interestingly, there was a modest but significant increase in total *MALAT1* expression suggesting a compensatory mechanism for the loss of intron 2 and loss of nuclear speckle localization (Figure S11B). We then tested the migration potential of these cells in transwell cell migration assays and found reduced cell migration in the intron 2-deleted clones, phenocopying the complete loss of *MALAT1* (Figures 7F and S11C). These data suggest that the retention of intron 2 is essential for *MALAT1* localization to nuclear speckles and it plays a key role in promoting cell migration.

## Discussion

Intron retention is a regulated process that shapes gene expression networks through mechanisms such as NMD and influences alternative protein isoform generation during differentiation, development, and disease.^16,58^ We previously identified a p53-induced lncRNA, *PURPL*, which exhibits growth-promoting functions, and that intron 2 of *PURPL* is retained in multiple cell lines.^43^ In this study, using an innovative RNA-coupled genome-wide CRISPR screen, we unexpectedly identified U2AF2 as a major inhibitor of intron 2 removal in *PURPL*. U2AF2 forms a heterodimer with U2AF1, binding to the Py-tract and the 3′ ss, respectively, to promote splicing by recruiting core spliceosomal components.^8,9,59^ Therefore, given the established role of U2AF2 as a critical positive regulator of splicing catalysis, we were surprised that U2AF2 inhibits the removal of *PURPL* intron 2. While U2AF1 was not identified as a hit in our screen, follow-up experiments revealed that U2AF1 also suppresses *PURPL* intron 2 removal, suggesting a previously unrecognized role for the U2AF complex as a negative regulator *PURPL* IR.

Beyond IR in *PURPL*, we sought to determine whether U2AF2 promotes IR in other transcripts. Using IR Finder,^54^ we identified other genes where U2AF2 promoted IR. One of the genes that drew our attention was the lncRNA *MALAT1* which showed two intron retention events regulated and bound by U2AF2. These introns contain weak splice sites suggesting that this may be the reason that they are regulated, and they are not constitutively spliced. Because *MALAT1* interacts with splicing factors within nuclear speckles^36^, we examined whether U2AF2 affects *MALAT1* localization to speckles. To determine whether this effect is directly linked to the retention of one or two of the introns or whether it is a secondary effect, we engineered cell lines expressing distinct *MALAT1* transcript isoforms. We observed that splicing is directly involved in the process and that intron 2 sequences are responsible for the inclusion of *MALAT1* in the nuclear speckles. We next tested the functional consequences of intron 2 retention in a breast cancer cell line by knocking out *MALAT1* intron 2 using CRISPR/Cas9. Clones in which intron 2 of *MALAT1* was deleted showed reduced cell migration, phenocopying *MALAT1*-null cells. It should be noted that although *MALAT1* transcripts lacking intron 2 whether expressed from constructs or endogenous intron 2 knockout, display predominantly diffused nucleoplasmic localization, this observation alone cannot distinguish whether the effect arises from a specific speckle-localization element embedded within intron 2 or from intron removal by the splicing process *per se*. Future studies involving mapping of the intronic sequences and targeted mutagenesis of the splice sites within *MALAT1* intron 2 could help distinguish whether speckle localization of *MALAT1* is inhibited by the splicing process itself or depends on a discrete element within the intron.

For *PURPL*, previous work established its context-dependent oncogenic activity^42,43^, but those studies depleted all transcripts from the *PURPL* locus despite the presence of multiple alternatively spliced isoforms. In the current study, overexpression of the intron 2-retaining *PURPL* isoform in CRISPRi-mediated *PURPL*-depleted cells increased proliferation, demonstrating isoform-specific oncogenic function. Together, these findings reveal that intron retention in both *MALAT1* and *PURPL* is a regulatory mechanism with direct consequences on lncRNA localization and cancer-related phenotypes.

The surprising finding of the non-canonical function of U2AF2 promoted us to determine the underlying mechanism. We first asked whether U2AF2 influences transcription initiation or elongation at the *PURPL* locus, given the tight coupling between transcription and splicing.^60,61^ Transcription kinetics can modulate IR^14,62^ and lncRNAs often lack proximal RNA-pol II phosphorylation over 5′ ss.^31^ We therefore assayed for PolII-pSer5 (transcription initiation) and PolII-pSer2 (transcription elongation) occupancy after U2AF2 knockdown using CUT&RUN-Seq but there was no significant difference around intron 2 or elsewhere across the *PURPL* locus (data not shown). We also examined the chromatin mark H3K36me3 which has been linked to IR^63^ and again observed no effect after U2AF2 depletion (data not shown).

U2AF2 functions by binding to the Py-tract, and the Py-tract of *PURPL* intron 2 and *MALAT1* introns 1 and 2 deviate from the consensus, containing a mix of pyrimidines and purines. These atypical sequences likely contribute to splicing inhibition as revealed by our data from the mini-gene reporter for *PURPL*. Given that U2AF1 and U2AF2 bind to multiple intronic sites, a plausible model is that their docking obstructs subsequent spliceosomal assembly. lncRNAs generally undergo less efficient splicing and consequently, they have higher levels of IR compared to protein coding genes.^30,31^ A study showed that lncRNA splicing strength correlates with 5′ ss strength and lncRNA Py-tract has higher thymidine content compared to protein coding genes.^64^ The reduced evolutionary pressure to maintain efficient splicing as they are not used as templates for protein synthesis, likely contributes to this pattern. This is also supported by their predominant nuclear localization.^25,26^

Our data shows that *PURPL* transcripts can be either nuclear or cytoplasmic, and U2AF2 regulation of IR offers a mechanism of nuclear detention. Similar to this notion, the lncRNA *TUG1* remains in the nucleus due to intron retention^65^, and the mouse lncRNA *pCharme* undergoes an IR event which renders it chromatin-bound through its association with MATR3 and PTBP1.^66^ Another aspect of the effects of IR lies in the turnover of the transcripts where additional sequences added by the introns may cause lower transcript stability. In support of this, nuclear lncRNAs have been shown to be less stable than cytoplasmic ones^27^, and *PURPL* also follows this rule. Notably, the human lncRNA *hFAST* is cytoplasmic and promotes pluripotency through WNT signaling, but the mouse homolog does not because of its nuclear localization.^67^

If U2AF2 is not required for intron removal in a subset of transcripts including those from the *PURPL* and *MALAT1* locus, how would the splicing occur? To address this question, we surveyed the literature for RNA-binding proteins known to facilitate splicing of transcripts with weak splice sites. This led us to SON, an RNA-binding protein reported to promote the splicing of transcripts that harbor suboptimal splice signals^68^ SON depletion leads to inefficient intron removal since it serves as a co-factor to promote splicing of weak splice sites, particularly in genes encoding for cell-cycle regulating proteins.^69^ Interestingly, SON knockdown increased intron-retention of RNAs interacting with nuclear speckles.^55^ We therefore investigated whether U2AF2 interacts with SON protein in *PURPL* and *MALAT1* intron removal regulation. Although SON depletion did not have any effect on IR, co-depletion of SON and U2AF2 restored *PURPL* and *MALAT1* intron retention. Notably, SON binding to *PURPL* and *MALAT1* increased in the absence of U2AF2 suggesting that U2AF2 inhibits the recruitment of SON for intron removal. Whether this functional interaction is accompanied by physical interaction of the two splicing regulators or mutual exclusive binding to *PURPL* remains to be tested. These observations point to a potential regulatory interplay between SON and U2AF2 in intron removal in *PURPL* and *MALAT1*, that will require more targeted mechanistic studies in future studies.

Introns have been proposed to act as docking elements that tether lncRNAs to chromatin, ^26,66^ and a recent study showed that nuclear speckles are associated with distinct classes of retained introns.^70^ This raises the question of what makes intron 2 important for speckle localization of *MALAT1*. One possibility is that this region recruits specific RBPs or adopts a structural configuration required for speckle association. Our data further indicates that intron 2 retention contributes to *MALAT1’s* role in cell migration, potentially because speckle localization is necessary to sustain this phenotype. Future studies are warranted to dig deeper into the underlying mechanism and to determine how intron retention shapes the function of *PURPL* and *MALAT1*.

Collectively, these findings reveal a non-canonical function of U2AF2 in promoting an intron retention program that governs the subcellular localization and function of the lncRNAs. As intron retention continues to emerge as a regulated form of alternative splicing, the evidence presented here expands the framework for the mechanisms and biological consequences of intron retention.

### Limitations of the study

Limitations of the study stem from the fact that the screen was conducted with an artificial system where only the sequences from *PURPL* intron 2 and its flanking exons were present in the construct. The system cannot capture regulatory elements outside the cloned regions or account for factors that influence splicing such as transcription and chromatin marks. In addition, although the library contained thousands of gRNAs, it is not exhaustive and may have missed additional regulators of *PURPL* intron 2 retention, such as U2AF1. Consistent with this, our data showed that U2AF2 knockdown did not completely abolish *PURPL* or *MALAT1* intron retention. Thus, intron retention in *PURPL* and *MALAT1* may be regulated by additional RNA-binding proteins in addition to U2AF2. In such a combinatorial regulatory context, depletion of U2AF2 alone would be expected to produce only a partial reduction in intron retention. Together, these observations support a significant and biologically meaningful role for U2AF2 in promoting intron retention in *PURPL* and *MALAT1* despite the incomplete penetrance of the knockdown.

## Materials and Methods

### Cell culture and treatment

HAP1, HCT116, HEK293T, HepG2, RPE1, SKHEP1, U2OS, WI38, and MDA-MB-231 cells were purchased from ATCC and maintained in Dulbecco’s Modified Eagle Medium (DMEM) (Gibco) supplemented with 10% (v/v) fetal bovine serum (Gibco) and 1% (v/v) PenStrep (Gibco) in a 5% CO_2_ atmosphere at 37°C. All cell lines were routinely confirmed to be free of mycoplasma using Venor GeM Mycoplasma Detection Kit (Millipore Sigma-Aldrich).

### CRASP-Seq *PURPL* screen

The *PURPL* minigene reporter, composed of exons 2 and 3 with the full native intronic sequence between them, was integrated into the CRASP-Seq genome-wide library. The *PURPL* intron 2 minigene reporter fragments were synthesized by TWIST Biosciences and cloned using NEBuilder into the Eco32I-digested CRASP-Seq knockout library vector as described previously.^48^ To ensure a minimum 250-fold library coverage, 16 Eco32I-digested vector reactions were prepared following the manufacturer's protocol, which included incubation at 50°C for 60 minutes. The reaction mixture was then precipitated with sodium acetate and ethanol, purified, and transformed into Endura competent cells (LGC Biosearch Technologies) by electroporation (1 mm cuvette, 25 μF, 200 Ω, 1,600 V). Colonies were plated on LB agar plates with 100 μg/mL carbenicillin. After overnight incubation at 30°C, bacterial colonies were pooled, and plasmids were extracted using an Endonuclease Free Plasmid Maxi Kit (QIAGEN).

Lentivirus was produced following established protocols.^48^ HEK293T cells (8 million per 15-cm plate) were transfected with a mixture containing 8 μg of the CRASP-Seq lentiviral library vector, 6.5 μg psPAX2 (Addgene #12260), 4 μg pMD2.G (Addgene #12260), 48 μL X-tremeGENE 9 DNA Transfection Reagent (Sigma-Aldrich), and 1.4 mL Opti-MEM (Gibco). After 24 hours, the medium was replaced with serum-free, high-BSA DMEM. Virus-containing medium was collected 48 hours later, centrifuged at 475 rcf for 5 minutes at 4°C, aliquoted, and stored at −80°C.

The lentiviral library was used to infect HAP1 and RPE1 cells expressing spCas9 and opCas12a nucleases.^48^ at a multiplicity of infection (MOI) of ~0.2. Infected cells were selected with 2 μg/mL puromycin for 48 hours, pooled, replated, and induced with doxycycline to activate reporter expression. Twenty-four hours post-induction, cells were harvested in aliquots of 10 million for downstream analyses.

Total RNA was extracted using the Qiagen RNeasy Plus Mini Kit, and polyadenylated mRNA was isolated with oligo(dT) Dynabeads (ThermoFisher Scientific). First-strand cDNA synthesis was performed with the Maxima H Minus First Strand cDNA Synthesis Kit and a custom primer annealing to the U6 promoter (ThermoFisher Scientific). PCR amplification targeted spliced and intron-containing transcripts using custom primers specific to exon 3 or intron 2, respectively, incorporating Illumina barcodes. PCR reactions were carried out with NEBNext Ultra II Q5 Master Mix (NEB), and products were resolved on a 1% agarose gel stained with SYBR Safe (ThermoFisher Scientific). Target bands were extracted and purified using a ThermoFisher gel extraction kit. Validated libraries were quantified using the Qubit dsDNA HS Assay (ThermoFisher Scientific) and the Agilent TapeStation system. Libraries were sequenced on the NovaSeq 6000 platform with a 25% PhiX spike-in and a 300-cycle kit. The sequencing strategy consisted of the following configuration: Read 1 (210 cycles), Index Read 1 (8 cycles), Index Read 2 (8 cycles) and Read 2 (85 cycles). Each sample yielded over 500 million total reads, ensuring robust data coverage.

## Data Analysis

A custom bioinformatics pipeline has been developed to analyze CRASP-Seq data. Cas9 and Cas12a guides, cell barcodes (CBCs), and unique molecular identifiers (UMIs) were retrieved using STAR (Dobin et al., 2013). SAMTools (Li et al., 2009) was used to manage sequencing alignment files. The aligned and annotated reads with all the necessary information including hybrid guide information, CBC, UMI, and splicing outcomes were deduplicated to carry out splicing regulation analysis only for complete and unique reads. Splicing reporter reads (R2) were analyzed for splicing outcomes by computing percent intron retention (PIR) and differential PIR (ΔPIR) as follows: PIR = [IE / (IE + EE)] × 100, where IE represents intron retention read counts, and EE represents exon-exon junction read counts.


$$•\;\Delta \text{PIR}={\text{PIR}}_{\text{guide}}-{\text{PIR}}_{\text{intergenic}}$$

The APIR for each gene and for each guide separately is shown in Table S1. To determine screen hits, a multi-step filtering process was employed to ensure precision and reliability in the results. Initially, guides with fewer than 10 reads were excluded from the analysis to eliminate low-confidence data. Next, a stringent ΔPIR threshold was established based on the intergenic guide distribution, with a False Discovery Rate (FDR) of 10%, to define statistically significant hits. This ΔPIR threshold was then applied at several levels of analysis. At the gene level, only genes meeting the threshold and with FPKM ≥ 0.1 were considered hits. For a gene to qualify, at least 50% of its associated guides had to pass the guide-level ΔPIR threshold. Additionally, a gene required a minimum of two guides to meet the criteria, which excluded instances where a single guide accounted for the signal. To classify genes as hits, they were required to meet the aforementioned threshold in both technical replicates, which were generated based on CBC-defined single-cell lineage groupings. All genes identified through combinatorial gene targeting that satisfied the previously described criteria were considered hits and they are shown in the ‘HITS’ tab of Table S1. The heatmap and Venn diagram in Figure 1C and 1E display single-gene hits as well as collapsed gene hits resulting from combinatorial targeting.

### eCLIP-seq data and functional enrichment analysis

eCLIP-seq data for U2AF2, U2AF1, PTBP1, PRPF8, and SRSF1 were obtained from the ENCODE database (encodeproject.org) with the following identifiers: ENCFF989JBA (CTRL), ENCFF566CFJ (CTRL), ENCFF536AFD (U2AF2), ENCFF913WRH (U2AF2), ENCFF159SPZ (U2AF2), ENCFF368XEI (U2AF2), ENCFF542NOA (U2AF1), ENCFF651VRQ (U2AF1), ENCFF363UDO (PTBP1), ENCFF044RKN (PRPF8), and ENCFF327NVE (SRSF1). For visualization of RNA-seq data, the Integrated Genome Viewer (IGV) version 17 was used. The functional enrichment analysis was performed using g:Profiler^71^ and the results are shown in the ‘gProfiler_Results’ tab in Table S1.

### siRNA transfections

For siRNA transfections, we used AllStars Negative Control siRNA (QIAGEN). U2AF2 siRNAs were purchased from Dharmacon (J-012380-18 and J-012380-19). U2AF1 siRNA was also purchased from Dharmacon (J-012325-10), as well as SON siRNAs (J-012983-05 and J-012983-06). Cells were reverse transfected with siRNA at a final concentration of 20 nM using Lipofectamine RNAiMAX (Invitrogen) in Opti-MEM I Reduced Serum Medium (Gibco) according to the manufacturer’s protocol. SKHEP1 cells were transfected for 72 hr before RNA harvesting. U2OS, HepG2 and WI38 cells were transfected for two rounds of 48 h + 72 h or 72 h + 72 h. For RNA FISH, SKHEP1, HCT116, and MDA-MB-231 cells were transfected for 72 hr. SKHEP1 expressing the *PURPL* Py-tract mutants were transfected for two rounds of 48 h and 24 h before harvesting, the cells were treated with 1μg/ml doxycycline to induce the transgene expression. For RNA-seq, the cells were transfected for 72 hr before harvested. For RNA stability assay, the cells were reseeded in 12-well plates 48 hr after siRNA transfection and 24 hr later they were treated with Act D for the indicated time points at 5ug/ml before RNA extraction and subsequent assays. For RNA-IP, the cells were transfected for 72 hr before harvested in 10 cm-plates.

### RNA extraction, RT-qPCR, RT-PCR, and splicing gels

Total RNA was extracted using TRIzol (Invitrogen) according to the manufacturer’s protocol. cDNA was then made using iScript^™^ Reverse Transcription Supermix (Bio-Rad). For real-time qPCR, all reactions were carried out on StepOnePlus real-time PCR System (Applied Biosystems) using FastStart SYBR Green Master Mix (Millipore Sigma). *GAPDH* mRNA was used to normalize expression except for RNA-IPs in Figure 2B where *18S* was used for normalization, and the relative expression/enrichment of RNAs was calculated using the 2^−ΔΔCt^ method. RT-qPCR primer sequences for each gene are indicated in Table S3. For splicing gels, cDNA was used to amplify the region flanking the retained intron with primers in the flanking exons with the addition of one primer inside the intron as indicated in Figure S1C. In the case of *MALAT1*, primers in the flanking exons were utilized due to the short length of introns. For assay of the effect of U2AF2 on the Py-tract mutants of *PURPL*, primers on the transgene were used instead of the endogenous *PURPL* exon 2 and 3. The sequences of the primers can be found in Table S5.

### RNA-IPs

For RNA-IP, two 10 cm-plates of SKHEP1 cells were lysed with RIPA buffer (Thermo Scientific) for 10 min on ice before spin down for 10 min at 4°C. The lysate was incubated with 1 μg of anti-U2AF2 (CST) or rabbit IgG (CST) in 500 μl total volume overnight at 4°C. The next day, 50 μl of Protein A/G Dynabeads (Pierce) were added to the mix and incubated for 2 hr at 4 °C. Beads were then washed 4 times with RIPA buffer and RNA was isolated using TRIzol. Equal volumes of extracted RNA were used for cDNA synthesis and RT-qPCR as described above. For siU2AF2-transfected cells, one 10 cm-plate was used, and all the lysate was incubated with SON antibody and not IgG.

### Immunoblotting

For immunoblotting, total cell lysate was prepared using RIPA buffer (Thermo Scientific) containing protease inhibitor cocktail (Roche). Protein concentration was determined using the BCA protein quantitation kit (Thermo Scientific). Cell lysate was loaded onto SDS-PAGE gel and transferred to a PVDF membrane (Thermo Scientific) using a semi-dry apparatus (Bio-Rad). The membrane was blocked in 5% skim milk (Millipore) in TBS containing 0.05% Tween 20. The following antibodies were used: anti-U2AF2 (1:1000, rabbit) (CST, 70471S), anti-GAPDH (1:10,000, rabbit) (CST, 2118L), anti-SON (1:500, rabbit) (Abcam, 121759), and secondary anti-rabbit (1:5000) (CST, 7074S).

### Nuclear cytoplasmic fractionation

For nuclear cytoplasmic fractionation, SKHEP1 cells were harvested after trypsinization at 80-90% confluence. Cells were pelleted and resuspended in 500 μL RSB buffer (10 mM Tris-HCl, 100 mM NaCl, and 2.5 mM MgCl2) containing 40 μg/mL digitonin (Invitrogen), and 100 U of RNase Out. After incubation on ice for 10 min, cell viability was tested with trypan blue to make sure that the cells were permeabilized. The cytoplasmic fraction was harvested as supernatant after centrifugation at 1,500 × g for 5 min at 4°C. The nuclear pellet was resuspended in 100ul RSB buffer. 100ul of the cytoplasmic fractionation were transferred to a fresh tube and 900ul of Trizol was added as well as to the nuclear extracts for RNA extraction and subsequent cDNA synthesis and RT-qPCR.

### Cell proliferation assay

The cell proliferation assay was conducted as previously described.^72^ To measure proliferation, parental SKHEP1 cells and *PURPL* CRISPRi cells overexpressing *PURPL* containing intron 2 were seeded at 1 x10^^5^ cells per well in 6-well plates in the presence of 1μg/ml doxycycline. After 3 days, the cell numbers and viability were measured. The cells were then passaged at 1x10^5 and measured again at 6 days and cumulative cell numbers were plotted.

### RNA-seq

The TruSeq Stranded mRNA libraries were pooled and sequenced on one NovaSeq6000 S1 flowcell using 2x101 cycles for pair-end run. The Real Time Analysis software (RTA v3.4.4) was used for processing raw data files, the Illumina bcl2fastq v2.20 was used to demultiplex and convert binary base calls and quality scores to fastq format. The sequencing reads were trimmed of adapters and low-quality bases using Cutadapt (v1.18). The trimmed reads were mapped to human reference genome (hg38) and Gencode annotation GENCODE_v30 GTF using STAR aligner (version 2.7.0f) with two-pass alignment option. RSEM (v1.3.1) was used for gene and transcript quantification based on GENCODE annotation file. Palantir Foundry was used for analysis of Differential Expression of Genes inside the secure NIH Integrated Data Analysis Platform (NIDAP). The DEG Analysis results are shown in Table S3.

### Iso-Seq

The libraries were prepared using PacBio standard Iso-seq library prep using SMRTbell prep kit 3.0. The libraries were sequenced on PacBio Sequel II platform using PacBio v2.0 chemistry. PacBio SMRTlink raw subreads were converted into HiFi circular consensus sequences (CCS) with minimum 3 passes. PacBio IsoSeq v3 pipeline was used to demultiplex the barcodes and remove primers. Additional refine steps included trimming poly-A tails and removing concatemers to generate Full Length Non-Concatemer (FLNC) reads. The FLNC reads were used to map to human reference (hg38) using Minimap2 software to generate alignment bam files. Hierarchical clustering and merging steps were performed to obtain consensus isoforms and the full-length (FL) consensus sequences. The high-quality FL transcripts were mapped to the reference genome by using the minimap2 software. Isoform classification and quality control were done using SQANTI3 software and Illumina short-read gene expression data was combined with PacBio Iso-seq for transcript quantification. The Iso-Seq Analysis results are shown in Table S4. The resulting bam files from the Iso-seq analysis were used for plotting and visualization with the R package Gviz (https://bioconductor.org/packages/release/bioc/html/Gviz.html).

### IRFinder

IRFinder^54^ was used to identify intron retention events that changed significantly on U2AF2 knockdown. A docker image was downloaded from Docker Hub (docker://cloxd/irfinder:2.0.1). The reference was built using pre-downloaded resources (IRFinder BuildRefProcess), using Ensembl release 92 and the 100bp mappability file provided by IRFinder. The Build-BED-refs.sh script was adjusted so that transcript biotype was not restricted to "processed_transcript" or "protein_coding". Intron retention was quantified using IRFinder in FastQ mode with adapter trimming. Differential intron retention events between siU2AF2 vs. siCTRL were identified with IRFinder Diff using DESeq2 with the default IR ratio ≥ 0.05 in at least one sample and no warning filters, which profiled 16,859 unique introns.

### Construct generation

For Py-tract mutants, gene fragments were purchased from Twist Biosciences and cloned into the pLCHKO-puro vector containing the WT sequences of *PURPL* (Figure 1A), between sites for NseI and AfeI. The *PURPL* transcript containing intron 2 was cloned into the pCW57 vector (Hygro) (Addgene #80922) in SalI and MluI sites. *MALAT1* transcripts were cloned into the pCW57 vector (BSD) (Addgene #80921) in SalI and MluI sites. The *PURPL* insert was generated by Twist Biosciences and the *MALAT1* sequence was amplified using Phusion DNA polymerase (NEB). For deletion mutants, we used the Q5^®^ Site-Directed Mutagenesis Kit (NEB). The primers used for cloning are listed in Table S5.

### Generation of *MALAT1* KO cell lines

For HCT116 *MALAT1* knockout, we used integration by non-homologous end joining, which was accomplished by introducing a simultaneous double-strand break in genomic DNA and in the targeting vectors (pCMV-Puro and pCMV-Hygro).^73,74^ Plasmids encoding spCas9 and sgRNAs were obtained from Addgene (Plasmids #41815 and #47108). Oligonucleotides for construction of sgRNAs were obtained from Integrated DNA Technologies, hybridized, phosphorylated and cloned into the sgRNA plasmid or targeting vector using BbsI sites.^75^ Target sequences for sgRNAs are provided in Table S5. These vectors were transfected into HCT116 using Lipofectamine 2000 following recommendations from the manufacturer. Three days after transfection, the cells were selected with Puromycin (0.5 μg/ml) and Hygromycin (100 μg/ml) to generate clonal populations. Genomic DNA from each clone was isolated using DNEasy Blood and Tissue Kit (Qiagen). PCRs to detect integration of the targeting vector at the target site were performed using KAPA2G Robust PCR kits (Kapa Biosystems) according to the manufacturer’s instructions. A typical reaction contained 20–100 ng of genomic DNA in Buffer A (5 μl), Enhancer (5 μl), dNTPs (0.5 μl), primers forward (PINCR Det FP, 1.25 μl) and reverse (Targeting vector Det RP, 1.25 μl) and KAPA2G Robust DNA Polymerase (0.5 U). The DNA sequences of the primers for each target are provided in Table S5. PCR products were visualized in 2% agarose gels and images were captured using a ChemiDoc-It2 (UVP).

To generate MDA-MB-231 whole-locus and intron 2 *MALAT1* knockout clones, we used the CRIPSR/Cas9 system by transfecting the cells with gRNAs and purified Cas9. 2 μl of each 30 μM gRNA was mixed with 1 μl (~5 μg) of purified Cas9 and 100 μl of 4D-Nucleofector^®^ X SE buffer SE from the Cell Line Kit (Lonza) supplemented with 18.5 μl of SE supplement per 100 μl total buffer volume. The mixture was incubated at room temperature for 10 min and 1x10^6 cells were resuspended in nucleofection cuvettes. Nucleofections were carried out using the 4D-Nucleofector^®^ System (Lonza). The cells were transferred to a 6-well plate, and they were single-cell sorted 48 h later. The clones were grown for 3-4 weeks before genomic DNA extraction and genotyping with PCR using the primers in Table S5.

### *PURPL* and *MALAT1* overexpression and transduction

*PURPL* intron 2 constructs were transduced in previously generated *PURPL* CRISPRi SKHEP1 cells.^43^ *MALAT1*-WT and deletion mutants were transduced in the two HCT116-*MALAT1* KO clones. For lentivirus generation, HEK293T cells were seeded at 2 × 10^5^ cells/well onto 6-well plates, and 1,200 ng of each construct was transfected with lentiviral package vectors using Lipofectamine 2000 (Life Technologies, Invitrogen) according to the manufacturer’s protocol. Virus-containing media were harvested at 48 and 72 h post-transfection and added to cells for 24 h. Transduced cells were selected with 500 μg/mL hygromycin for 2 weeks (SKHEP1) or 10 μ g/ml Blasticidin (HCT116).

### RNA FISH, IF and Quantification of FISH

The *PURPL* intron probe set was custom-designed using the Stellaris Probe Designer, tagged with Quasar 570 dye and purchased from Stellaris. The *MALAT1* smFISH probe set with Quasar 670 dye was purchased from Stellaris (Cat# VSMF-2211-5). For *PURPL* intron smRNA-FISH, SKHEP1 cells were seeded on coverslips and cultured for 24 hours at 37°C with 5% CO_2_ before inducing DNA damage by incubating the cells with 2 mM hydroxyurea for 24 hours (or regular growth media for control). For *MALAT1* smRNA-FISH, HCT116 and MDA-MB-231 *MALAT1* KO cells were seeded on coverslips and cultured for 24 hours at 37°C with 5% CO_2_. *MALAT1* WT, *MALAT1* del-1, *MALAT1* del-2, *MALAT1* del1+2, or empty vector expression was induced by incubating the cells with 1 μg/mL doxycycline for 48 hours. The cells that were transfected with siRNA for U2AF2 were seeded on coverslips at the time of transfection.Cells were fixed with 4% PFA for 15 min at room temperature, permeabilized with 0.5% Triton X-100, and washed with washing buffer (10% formamide, 2XSSC) for 5 min. Probe was added to hybridization buffer (10% formamide in Stellaris hybridization buffer Cat# SMF-HB1-10) at a final concentration of 125 nM. Hybridization was done in a humidified chamber in the dark overnight at 37°C. After hybridization, the coverslips were washed twice with wash buffer for 30 min at 37°C and then post-fixed with 4% PFA for 10 min at room temperature. The coverslips were blocked with 5% normal goat serum and then incubated with SON antibody (Sigma Cat# HPA023535). Primary and secondary antibodies were diluted in 1% normal goat serum. Antibody incubations were done at room temperature for 1 hour and washed with PBS. DNA was counterstained by DAPI. The coverslips were then washed with 4XSSC for 5 min at room temperature and mounted in VectaShield Antifade Mounting Medium (Vector Laboratories). Images were taken using DeltaVision microscope (GE Healthcare) equipped with 60 X/1.42 NA oil immersion objective (Olympus) and CoolSNAP-HQ2 camera. To quantify the speckle to nucleoplasm signal intensity ratio of *MALAT1* in Figures 5B, 7B, 7D, and S8B ImageJ was used to create nucleus masks based on DAPI and speckles mask based on SON. The signal intensity of *MALAT1* in the speckles mask was divided by the *MALAT1* signal intensity in the nucleus-minus-speckles area. For the quantification of Figures 6B, 7E and S10B, the cells with *MALAT1* FISH signal showing prominent speckle patterns similar to SON were classified as "Speckle" pattern, and the cells with a diffused *MALAT1* FISH signal were classified as "Diffused". Only *MALAT1*-positive cells were counted for quantification.

### Transwell cell migration assays

For the transwell assay, WT and KO MDA-MB-231 clones were seeded at 10 x 10^5 cells per well in 12-well plates. 24 hr later, the media were removed and replaced with media containing 1% FBS. After 5 hr, the cells were trypsinized and seeded in the top chamber of the transwell plates (Corning, #354578) at 25,000 cells per well at a volume of 100 μl. The lower chamber of the transwell is filled with 700 μl of media containing 10% FBS. The cells were incubated for 24 hr and the transwell were removed from the plates. The non-migrated cells were scraped with a cotton swab and the migrated cells were fixed with ice-cold methanol for 5 min and stained with 0.05% crystal violet blue. The migrated cells were counted under a light microscope.

### Statistics

Statistics were performed using the Student’s t-test.

## Supplementary Material

Supplement 1

## Data Availability

The Iso-Seq data files have been deposited at GEO with accession number GSE288684 and the RNA-Seq data files have been deposited at GEO with accession number GSE288685.

## References

[1] Wilkinson M.E., Charenton C., and Nagai K. (2020). RNA Splicing by the Spliceosome. Annu Rev Biochem 89, 359–388. 10.1146/annurev-biochem-091719-064225. 31794245

[2] Marasco L.E., and Kornblihtt A.R. (2023). The physiology of alternative splicing. Nat Rev Mol Cell Biol 24, 242–254. 10.1038/s41580-022-00545-z. 36229538

[3] Matera A.G., and Wang Z. (2014). A day in the life of the spliceosome. Nat Rev Mol Cell Biol 15, 108–121. 10.1038/nrm3742. 24452469 PMC4060434

[4] Black D.L. (2003). Mechanisms of alternative pre-messenger RNA splicing. Annu Rev Biochem 72, 291–336. 10.1146/annurev.biochem.72.121801.161720. 12626338

[5] Ruskin B., Zamore P.D., and Green M.R. (1988). A factor, U2AF, is required for U2 snRNP binding and splicing complex assembly. Cell 52, 207–219. 10.1016/0092-8674(88)90509-0. 2963698

[6] Berglund J.A., Chua K., Abovich N., Reed R., and Rosbash M. (1997). The splicing factor BBP interacts specifically with the pre-mRNA branchpoint sequence UACUAAC. Cell 89, 781–787. 10.1016/s0092-8674(00)80261-5. 9182766

[7] Berglund J.A., Abovich N., and Rosbash M. (1998). A cooperative interaction between U2AF65 and mBBP/SF1 facilitates branchpoint region recognition. Genes Dev 12, 858–867. 10.1101/gad.12.6.858. 9512519 PMC316625

[8] Wu S., Romfo C.M., Nilsen T.W., and Green M.R. (1999). Functional recognition of the 3' splice site AG by the splicing factor U2AF35. Nature 402, 832–835. 10.1038/45590. 10617206

[9] Zorio D.A., and Blumenthal T. (1999). Both subunits of U2AF recognize the 3' splice site in Caenorhabditis elegans. Nature 402, 835–838. 10.1038/45597. 10617207

[10] Wang E.T., Sandberg R., Luo S., Khrebtukova I., Zhang L., Mayr C., Kingsmore S.F., Schroth G.P., and Burge C.B. (2008). Alternative isoform regulation in human tissue transcriptomes. Nature 456, 470–476. 10.1038/nature07509. 18978772 PMC2593745

[11] Nilsen T.W., and Graveley B.R. (2010). Expansion of the eukaryotic proteome by alternative splicing. Nature 463, 457–463. 10.1038/nature08909. 20110989 PMC3443858

[12] Pan Q., Shai O., Lee L.J., Frey B.J., and Blencowe B.J. (2008). Deep surveying of alternative splicing complexity in the human transcriptome by high-throughput sequencing. Nat Genet 40, 1413–1415. 10.1038/ng.259. 18978789

[13] Keren H., Lev-Maor G., and Ast G. (2010). Alternative splicing and evolution: diversification, exon definition and function. Nat Rev Genet 11, 345–355. 10.1038/nrg2776. 20376054

[14] Braunschweig U., Barbosa-Morais N.L., Pan Q., Nachman E.N., Alipanahi B., Gonatopoulos-Pournatzis T., Frey B., Irimia M., and Blencowe B.J. (2014). Widespread intron retention in mammals functionally tunes transcriptomes. Genome Res 24, 1774–1786. 10.1101/gr.177790.114. 25258385 PMC4216919

[15] Wong J.J., Ritchie W., Ebner O.A., Selbach M., Wong J.W., Huang Y., Gao D., Pinello N., Gonzalez M., Baidya K., (2013). Orchestrated intron retention regulates normal granulocyte differentiation. Cell 154, 583–595. 10.1016/j.cell.2013.06.052. 23911323

[16] Monteuuis G., Wong J.J.L., Bailey C.G., Schmitz U., and Rasko J.E.J. (2019). The changing paradigm of intron retention: regulation, ramifications and recipes. Nucleic Acids Res 47, 11497–11513. 10.1093/nar/gkz1068. 31724706 PMC7145568

[17] Eom T., Zhang C., Wang H., Lay K., Fak J., Noebels J.L., and Darnell R.B. (2013). NOVA-dependent regulation of cryptic NMD exons controls synaptic protein levels after seizure. Elife 2, e00178. 10.7554/eLife.00178. 23359859 PMC3552424

[18] Hossain M.A., Claggett J.M., Edwards S.R., Shi A., Pennebaker S.L., Cheng M.Y., Hasty J., and Johnson T.L. (2016). Posttranscriptional Regulation of Gcr1 Expression and Activity Is Crucial for Metabolic Adjustment in Response to Glucose Availability. Mol Cell 62, 346–358. 10.1016/j.molcel.2016.04.012. 27153533 PMC5117908

[19] Cha S., Hong C.P., Kang H.A., and Hahn J.S. (2021). Differential activation mechanisms of two isoforms of Gcr1 transcription factor generated from spliced and un-spliced transcripts in Saccharomyces cerevisiae. Nucleic Acids Res 49, 745–759. 10.1093/nar/gkaa1221. 33367825 PMC7826247

[20] Schmitz U., Pinello N., Jia F., Alasmari S., Ritchie W., Keightley M.C., Shini S., Lieschke G.J., Wong J.J., and Rasko J.E.J. (2017). Intron retention enhances gene regulatory complexity in vertebrates. Genome Biol 18, 216. 10.1186/s13059-017-1339-3. 29141666 PMC5688624

[21] Dvinge H., and Bradley R.K. (2015). Widespread intron retention diversifies most cancer transcriptomes. Genome Med 7, 45. 10.1186/s13073-015-0168-9. 26113877 PMC4480902

[22] Boutz P.L., Bhutkar A., and Sharp P.A. (2015). Detained introns are a novel, widespread class of post-transcriptionally spliced introns. Genes Dev 29, 63–80. 10.1101/gad.247361.114. 25561496 PMC4281565

[23] Quinn J.J., and Chang H.Y. (2016). Unique features of long non-coding RNA biogenesis and function. Nat Rev Genet 17, 47–62. 10.1038/nrg.2015.10. 26666209

[24] Ulitsky I., and Bartel D.P. (2013). lincRNAs: genomics, evolution, and mechanisms. Cell 154, 26–46. 10.1016/j.cell.2013.06.020. 23827673 PMC3924787

[25] Guo C.J., Xu G., and Chen L.L. (2020). Mechanisms of Long Noncoding RNA Nuclear Retention. Trends Biochem Sci 45, 947–960. 10.1016/j.tibs.2020.07.001. 32800670

[26] Zuckerman B., and Ulitsky I. (2019). Predictive models of subcellular localization of long RNAs. RNA 25, 557–572. 10.1261/rna.068288.118. 30745363 PMC6467007

[27] Clark M.B., Johnston R.L., Inostroza-Ponta M., Fox A.H., Fortini E., Moscato P., Dinger M.E., and Mattick J.S. (2012). Genome-wide analysis of long noncoding RNA stability. Genome Res 22, 885–898. 10.1101/gr.131037.111. 22406755 PMC3337434

[28] Cabili M.N., Dunagin M.C., McClanahan P.D., Biaesch A., Padovan-Merhar O., Regev A., Rinn J.L., and Raj A. (2015). Localization and abundance analysis of human lncRNAs at single-cell and single-molecule resolution. Genome Biol 16, 20. 10.1186/s13059-015-0586-4. 25630241 PMC4369099

[29] Tilgner H., Knowles D.G., Johnson R., Davis C.A., Chakrabortty S., Djebali S., Curado J., Snyder M., Gingeras T.R., and Guigo R. (2012). Deep sequencing of subcellular RNA fractions shows splicing to be predominantly co-transcriptional in the human genome but inefficient for lncRNAs. Genome Res 22, 1616–1625. 10.1101/gr.134445.111. 22955974 PMC3431479

[30] Mele M., Mattioli K., Mallard W., Shechner D.M., Gerhardinger C., and Rinn J.L. (2017). Chromatin environment, transcriptional regulation, and splicing distinguish lincRNAs and mRNAs. Genome Res 27, 27–37. 10.1101/gr.214205.116. 27927715 PMC5204342

[31] Mukherjee N., Calviello L., Hirsekorn A., de Pretis S., Pelizzola M., and Ohler U. (2017). Integrative classification of human coding and noncoding genes through RNA metabolism profiles. Nat Struct Mol Biol 24, 86–96. 10.1038/nsmb.3325. 27870833

[32] Gutschner T., Hammerle M., Eissmann M., Hsu J., Kim Y., Hung G., Revenko A., Arun G., Stentrup M., Gross M., (2013). The noncoding RNA MALAT1 is a critical regulator of the metastasis phenotype of lung cancer cells. Cancer Res 73, 1180–1189. 10.1158/0008-5472.CAN-12-2850. 23243023 PMC3589741

[33] Hutchinson J.N., Ensminger A.W., Clemson C.M., Lynch C.R., Lawrence J.B., and Chess A. (2007). A screen for nuclear transcripts identifies two linked noncoding RNAs associated with SC35 splicing domains. BMC Genomics 8, 39. 10.1186/1471-2164-8-39. 17270048 PMC1800850

[34] Ji P., Diederichs S., Wang W., Boing S., Metzger R., Schneider P.M., Tidow N., Brandt B., Buerger H., Bulk E., (2003). MALAT-1, a novel noncoding RNA, and thymosin beta4 predict metastasis and survival in early-stage non-small cell lung cancer. Oncogene 22, 8031–8041. 10.1038/sj.onc.1206928. 12970751

[35] Bernard D., Prasanth K.V., Tripathi V., Colasse S., Nakamura T., Xuan Z., Zhang M.Q., Sedel F., Jourdren L., Coulpier F., (2010). A long nuclear-retained non-coding RNA regulates synaptogenesis by modulating gene expression. EMBO J 29, 3082–3093. 10.1038/emboj.2010.199. 20729808 PMC2944070

[36] Tripathi V., Ellis J.D., Shen Z., Song D.Y., Pan Q., Watt A.T., Freier S.M., Bennett C.F., Sharma A., Bubulya P.A., (2010). The nuclear-retained noncoding RNA MALAT1 regulates alternative splicing by modulating SR splicing factor phosphorylation. Mol Cell 39, 925–938. 10.1016/j.molcel.2010.08.011. 20797886 PMC4158944

[37] Shinn M.K., Tomares D.T., Liu V., Pant A., Qiu Y., Vitalis A., Song Y.J., Ayala Y., Ruff K.M., Strout G.W., (2026). Nuclear speckle proteins form intrinsic and MALAT1-dependent microphases. Cell 189, 832–852 e824. 10.1016/j.cell.2025.11.026. 41421357 PMC12922802

[38] Spector D.L., and Lamond A.I. (2011). Nuclear speckles. Cold Spring Harb Perspect Biol 3. 10.1101/cshperspect.a000646. PMC3039535 20926517

[39] Sharma A., Takata H., Shibahara K., Bubulya A., and Bubulya P.A. (2010). Son is essential for nuclear speckle organization and cell cycle progression. Mol Biol Cell 21, 650–663. 10.1091/mbc.e09-02-0126. 20053686 PMC2820428

[40] Nakagawa S., Ip J.Y., Shioi G., Tripathi V., Zong X., Hirose T., and Prasanth K.V. (2012). Malat1 is not an essential component of nuclear speckles in mice. RNA 18, 1487–1499. 10.1261/rna.033217.112. 22718948 PMC3404370

[41] Zhang B., Arun G., Mao Y.S., Lazar Z., Hung G., Bhattacharjee G., Xiao X., Booth C.J., Wu J., Zhang C., and Spector D.L. (2012). The lncRNA Malat1 is dispensable for mouse development but its transcription plays a cis-regulatory role in the adult. Cell Rep 2, 111–123. 10.1016/j.celrep.2012.06.003. 22840402 PMC3408587

[42] Li X.L., Subramanian M., Jones M.F., Chaudhary R., Singh D.K., Zong X., Gryder B., Sindri S., Mo M., Schetter A., (2017). Long Noncoding RNA PURPL Suppresses Basal p53 Levels and Promotes Tumorigenicity in Colorectal Cancer. Cell Rep 20, 2408–2423. 10.1016/j.celrep.2017.08.041. 28877474 PMC5777516

[43] Hartford C.C.R., Shrestha R.L., Pongor L., Zhao Y., Chen X., Fromont C., Chaudhary R., Li X.L., Pasterczyk K.R., Kumar R., (2022). Context-Dependent Function of Long Noncoding RNA PURPL in Transcriptome Regulation during p53 Activation. Mol Cell Biol 42, e0028922. 10.1128/mcb.00289-22. 36342127 PMC9753727

[44] Behera A.K., Kim J.J., Kordale S., Pekovic F., Damodaran A.P., Kumari B., Vidak S., Dickson E., Xiao M.S., Duncan G., (2025). RNA-coupled CRISPR screens reveal ZNF207 as a regulator of LMNA aberrant splicing in progeria. Mol Cell. 10.1016/j.molcel.2025.12.003. PMC12952242 41475346

[45] Aregger M., Xing K., and Gonatopoulos-Pournatzis T. (2021). Application of CHyMErA Cas9-Cas12a combinatorial genome-editing platform for genetic interaction mapping and gene fragment deletion screening. Nat Protoc 16, 4722–4765. 10.1038/s41596-021-00595-1. 34508260 PMC12314557

[46] Gier R.A., Budinich K.A., Evitt N.H., Cao Z., Freilich E.S., Chen Q., Qi J., Lan Y., Kohli R.M., and Shi J. (2020). High-performance CRISPR-Cas12a genome editing for combinatorial genetic screening. Nat Commun 11, 3455. 10.1038/s41467-020-17209-1. 32661245 PMC7359328

[47] Gonatopoulos-Pournatzis T., Aregger M., Brown K.R., Farhangmehr S., Braunschweig U., Ward H.N., Ha K.C.H., Weiss A., Billmann M., Durbic T., (2020). Genetic interaction mapping and exon-resolution functional genomics with a hybrid Cas9-Cas12a platform. Nat Biotechnol 38, 638–648. 10.1038/s41587-020-0437-z. 32249828

[48] Xiao M.S., Damodaran A.P., Kumari B., Dickson E., Xing K., On T.A., Parab N., King H.E., Perez A.R., Guiblet W.M., (2024). Genome-scale exon perturbation screens uncover exons critical for cell fitness. Mol Cell 84, 2553–2572 e2519. 10.1016/j.molcel.2024.05.024. 38917794 PMC11246229

[49] Van Nostrand E.L., Pratt G.A., Shishkin A.A., Gelboin-Burkhart C., Fang M.Y., Sundararaman B., Blue S.M., Nguyen T.B., Surka C., Elkins K., (2016). Robust transcriptome-wide discovery of RNA-binding protein binding sites with enhanced CLIP (eCLIP). Nat Methods 13, 508–514. 10.1038/nmeth.3810. 27018577 PMC4887338

[50] Consortium E.P. (2012). An integrated encyclopedia of DNA elements in the human genome. Nature 489, 57–74. 10.1038/nature11247. 22955616 PMC3439153

[51] Luo Y., Hitz B.C., Gabdank I., Hilton J.A., Kagda M.S., Lam B., Myers Z., Sud P., Jou J., Lin K., (2020). New developments on the Encyclopedia of DNA Elements (ENCODE) data portal. Nucleic Acids Res 48, D882–D889. 10.1093/nar/gkz1062. 31713622 PMC7061942

[52] Berhane T., Holm A., Karstensen K.T., Petri A., Ilieva M.S., Krarup H., Vyberg M., Lovendorf M.B., and Kauppinen S. (2022). Knockdown of the long noncoding RNA PURPL induces apoptosis and sensitizes liver cancer cells to doxorubicin. Sci Rep 12, 19502. 10.1038/s41598-022-23802-9. 36376362 PMC9663437

[53] Han S., Li X., Wang K., Zhu D., Meng B., Liu J., Liang X., Jin Y., Liu X., Wen Q., and Zhou L. (2021). PURPL represses autophagic cell death to promote cutaneous melanoma by modulating ULK1 phosphorylation. Cell Death Dis 12, 1070. 10.1038/s41419-021-04362-8. 34759263 PMC8581000

[54] Middleton R., Gao D., Thomas A., Singh B., Au A., Wong J.J., Bomane A., Cosson B., Eyras E., Rasko J.E., and Ritchie W. (2017). IRFinder: assessing the impact of intron retention on mammalian gene expression. Genome Biol 18, 51. 10.1186/s13059-017-1184-4. 28298237 PMC5353968

[55] Wu J., Xiao Y., Liu Y., Wen L., Jin C., Liu S., Paul S., He C., Regev O., and Fei J. (2024). Dynamics of RNA localization to nuclear speckles are connected to splicing efficiency. Sci Adv 10, eadp7727. 10.1126/sciadv.adp7727. 39413186 PMC11482332

[56] Martinez-Terroba E., Plasek-Hegde L.M., Chiotakakos I., Li V., de Miguel F.J., Robles-Oteiza C., Tyagi A., Politi K., Zamudio J.R., and Dimitrova N. (2024). Overexpression of Malat1 drives metastasis through inflammatory reprogramming of the tumor microenvironment. Sci Immunol 9, eadh5462. 10.1126/sciimmunol.adh5462. 38875320 PMC12087577

[57] Arun G., Aggarwal D., and Spector D.L. (2020). MALAT1 Long Non-Coding RNA: Functional Implications. Noncoding RNA 6. 10.3390/ncrna6020022. PMC7344863 32503170

[58] Jacob A.G., and Smith C.W.J. (2017). Intron retention as a component of regulated gene expression programs. Hum Genet 136, 1043–1057. 10.1007/s00439-017-1791-x. 28391524 PMC5602073

[59] Shao C., Yang B., Wu T., Huang J., Tang P., Zhou Y., Zhou J., Qiu J., Jiang L., Li H., (2014). Mechanisms for U2AF to define 3' splice sites and regulate alternative splicing in the human genome. Nat Struct Mol Biol 21, 997–1005. 10.1038/nsmb.2906. 25326705 PMC4429597

[60] Naftelberg S., Schor I.E., Ast G., and Kornblihtt A.R. (2015). Regulation of alternative splicing through coupling with transcription and chromatin structure. Annu Rev Biochem 84, 165–198. 10.1146/annurev-biochem-060614-034242. 26034889

[61] Herzel L., Ottoz D.S.M., Alpert T., and Neugebauer K.M. (2017). Splicing and transcription touch base: co-transcriptional spliceosome assembly and function. Nat Rev Mol Cell Biol 18, 637–650. 10.1038/nrm.2017.63. 28792005 PMC5928008

[62] Wong J.J., Gao D., Nguyen T.V., Kwok C.T., van Geldermalsen M., Middleton R., Pinello N., Thoeng A., Nagarajah R., Holst J., (2017). Intron retention is regulated by altered MeCP2-mediated splicing factor recruitment. Nat Commun 8, 15134. 10.1038/ncomms15134. 28480880 PMC5424149

[63] Guo R., Zheng L., Park J.W., Lv R., Chen H., Jiao F., Xu W., Mu S., Wen H., Qiu J., (2014). BS69/ZMYND11 reads and connects histone H3.3 lysine 36 trimethylation-decorated chromatin to regulated pre-mRNA processing. Mol Cell 56, 298–310. 10.1016/j.molcel.2014.08.022. 25263594 PMC4363072

[64] Krchnakova Z., Thakur P.K., Krausova M., Bieberstein N., Haberman N., Muller-McNicoll M., and Stanek D. (2019). Splicing of long non-coding RNAs primarily depends on polypyrimidine tract and 5' splice-site sequences due to weak interactions with SR proteins. Nucleic Acids Res 47, 911–928. 10.1093/nar/gky1147. 30445574 PMC6344860

[65] Dumbovic G., Braunschweig U., Langner H.K., Smallegan M., Biayna J., Hass E.P., Jastrzebska K., Blencowe B., Cech T.R., Caruthers M.H., and Rinn J.L. (2021). Nuclear compartmentalization of TERT mRNA and TUG1 lncRNA is driven by intron retention. Nat Commun 12, 3308. 10.1038/s41467-021-23221-w. 34083519 PMC8175569

[66] Desideri F., Cipriano A., Petrezselyova S., Buonaiuto G., Santini T., Kasparek P., Prochazka J., Janson G., Paiardini A., Calicchio A., (2020). Intronic Determinants Coordinate Charme lncRNA Nuclear Activity through the Interaction with MATR3 and PTBP1. Cell Rep 33, 108548. 10.1016/j.celrep.2020.108548. 33357424 PMC7773549

[67] Guo C.J., Ma X.K., Xing Y.H., Zheng C.C., Xu Y.F., Shan L., Zhang J., Wang S., Wang Y., Carmichael G.G., (2020). Distinct Processing of lncRNAs Contributes to Non-conserved Functions in Stem Cells. Cell 181, 621–636 e622. 10.1016/j.cell.2020.03.006. 32259487

[68] Sharma A., Markey M., Torres-Munoz K., Varia S., Kadakia M., Bubulya A., and Bubulya P.A. (2011). Son maintains accurate splicing for a subset of human pre mRNAs. J Cell Sci 124, 4286–4298. 10.1242/jcs.092239. 22193954 PMC3258111

[69] Ahn E.Y., DeKelver R.C., Lo M.C., Nguyen T.A., Matsuura S., Boyapati A., Pandit S., Fu X.D., and Zhang D.E. (2011). SON controls cell-cycle progression by coordinated regulation of RNA splicing. Mol Cell 42, 185–198. 10.1016/j.molcel.2011.03.014. 21504830 PMC3137374

[70] Barutcu A.R., Wu M., Braunschweig U., Dyakov B.J.A., Luo Z., Turner K.M., Durbic T., Lin Z.Y., Weatheritt R.J., Maass P.G., (2022). Systematic mapping of nuclear domain-associated transcripts reveals speckles and lamina as hubs of functionally distinct retained introns. Mol Cell 82, 1035–1052 e1039. 10.1016/j.molcel.2021.12.010. 35182477

[71] Kolberg L., Raudvere U., Kuzmin I., Adler P., Vilo J., and Peterson H. (2023). g:Profiler-interoperable web service for functional enrichment analysis and gene identifier mapping (2023 update). Nucleic Acids Res 51, W207–W212. 10.1093/nar/gkad347. 37144459 PMC10320099

[72] Lee J.S., Dan T., Zhang H., Cheng Y., Rehfeld F., Brugarolas J., and Mendell J.T. (2025). An ultraconserved snoRNA-like element in long noncoding RNA CRNDE promotes ribosome biogenesis and cell proliferation. Mol Cell 85, 1543–1560 e1510. 10.1016/j.molcel.2025.03.006. 40185099 PMC12009208

[73] Brown A., Winter J., Gapinske M., Tague N., Woods W.S., and Perez-Pinera P. (2019). Multiplexed and tunable transcriptional activation by promoter insertion using nuclease-assisted vector integration. Nucleic Acids Res 47, e67. 10.1093/nar/gkz210. 30931472 PMC6614798

[74] Brown A., Woods W.S., and Perez-Pinera P. (2016). Multiplexed Targeted Genome Engineering Using a Universal Nuclease-Assisted Vector Integration System. ACS Synth Biol 5, 582–588. 10.1021/acssynbio.6b00056. 27159246

[75] Brown A., Woods W.S., and Perez-Pinera P. (2017). Targeted Gene Activation Using RNA-Guided Nucleases. Methods Mol Biol 1468, 235–250. 10.1007/978-1-4939-4035-616. 27662880

